# ZNRF1 Mediates Epidermal Growth Factor Receptor Ubiquitination to Control Receptor Lysosomal Trafficking and Degradation

**DOI:** 10.3389/fcell.2021.642625

**Published:** 2021-04-29

**Authors:** Chia-Hsing Shen, Chih-Chang Chou, Ting-Yu Lai, Jer-En Hsu, You-Sheng Lin, Huai-Yu Liu, Yan-Kai Chen, I-Lin Ho, Pang-Hung Hsu, Tsung-Hsien Chuang, Chih-Yuan Lee, Li-Chung Hsu

**Affiliations:** ^1^Institute of Molecular Medicine, National Taiwan University, Taipei, Taiwan; ^2^Department of Bioscience and Biotechnology, National Taiwan Ocean University, Keelung City, Taiwan; ^3^Immunology Research Center, National Health Research Institutes, Zhunan, Taiwan; ^4^Department of Surgery, National Taiwan University Hospital, Taipei, Taiwan; ^5^Center of Precision Medicine, College of Medicine, National Taiwan University, Taipei, Taiwan

**Keywords:** ZNRF1, epidermal growth factor receptor (EGFR), ubiquitination, lysosomal trafficking, herpes simplex virus 1 (HSV-1)

## Abstract

Activation of the epidermal growth factor receptor (EGFR) is crucial for development, tissue homeostasis, and immunity. Dysregulation of EGFR signaling is associated with numerous diseases. EGFR ubiquitination and endosomal trafficking are key events that regulate the termination of EGFR signaling, but their underlying mechanisms remain obscure. Here, we reveal that ZNRF1, an E3 ubiquitin ligase, controls ligand-induced EGFR signaling via mediating receptor ubiquitination. Deletion of ZNRF1 inhibits endosome-to-lysosome sorting of EGFR, resulting in delayed receptor degradation and prolonged downstream signaling. We further demonstrate that ZNRF1 and Casitas B-lineage lymphoma (CBL), another E3 ubiquitin ligase responsible for EGFR ubiquitination, mediate ubiquitination at distinct lysine residues on EGFR. Furthermore, loss of ZNRF1 results in increased susceptibility to herpes simplex virus 1 (HSV-1) infection due to enhanced EGFR-dependent viral entry. Our findings identify ZNRF1 as a novel regulator of EGFR signaling, which together with CBL controls ligand-induced EGFR ubiquitination and lysosomal trafficking.

## Introduction

The epidermal growth factor receptor (EGFR) plays crucial roles in numerous cellular functions required for development and tissues homeostasis, including cell growth, proliferation, differentiation, and migration (Schlessinger, 2002). Binding of growth factors, such as epidermal growth factor (EGF), to the extracellular region of EGFR induces receptor dimerization and tyrosine kinase activation, resulting in its autophosphorylation. The phosphorylated tyrosine residues on the carboxy-terminus of EGFR serve as docking sites that recruit various adaptor proteins containing Src Homology 2 (SH2) or phosphotyrosine binding (PTB) domains, which further induces the activation of multiple downstream signaling pathways involved in distinct cellular functions (Lemmon et al., 2014). These signaling pathways include the phosphatidylinositol 3-kinase (PI3K)/AKT (Soltoff and Cantley, 1996), Ras/mitogen-activated kinase (MAPK) (Hallberg et al., 1994), mammalian target of rapamycin complex 1/p70 S6 kinase (mTORC1-S6K) (Fan et al., 2009), and phospholipase C-γ pathways (Wahl et al., 1990). EGFR signaling has recently been shown to participate in innate immune signaling including Toll-like receptors (TLRs) to promote host defense against pathogenic infection (Yamashita et al., 2012; Chattopadhyay et al., 2015). In contrast, viruses such as HSV-1 and vaccinia virus subvert EGFR signaling to facilitate their infection (Zheng et al., 2014; Beerli et al., 2019). Thus, EGFR expression and signaling must be tightly regulated. Aberrant EGFR activation often leads to the progression of various diseases and cancers (Du and Lovly, 2018).

Endocytic trafficking of EGFR is a key mechanism for regulating EGFR signaling (Madshus and Stang, 2009; Tomas et al., 2014). Upon EGF stimulation, activated EGFR is immediately internalized into the early endosomes, where it continues to transmit signals (Vieira et al., 1996; Brankatschk et al., 2012; Sousa et al., 2012). Endosomal EGFR is either recycled back to the cell surface (Sorkin et al., 1991), translocated to the nucleus (Demory et al., 2009; Wang et al., 2010), or trafficked to multivesicular bodies (MVBs)/lysosomes for degradation. Thereby, the sorting and lysosomal degradation of activated EGFR are important mechanisms for terminating EGFR signaling. EGFR mutants found in tumor patients are not internalized or transported to the MVBs/lysosomes, resulting in enhanced and prolonged activation of EGFR and its downstream MAPK signaling that is essential for tumor cell proliferation and invasion (Huang et al., 2006; Goh et al., 2010).

Accumulating evidence revealed that ubiquitination serves as a critical sorting signal for endocytic trafficking of EGFR (Clague et al., 2012). EGF engagement induces rapid ubiquitination of EGFR on lysine residues within its tyrosine kinase domain (TKD) (Stang et al., 2000; Huang et al., 2006). The endosomal complex required for transport (ESCRT) machinery then recognizes the ubiquitinated EGFR and sort the receptor into intraluminal vesicles of the MVBs for subsequent lysosomal degradation (Raiborg and Stenmark, 2009; Henne et al., 2011). Hepatocyte growth factor-regulated tyrosine kinase substrate (HRS), a component of the ESCRT-0 complex, first recognizes ubiquitinated EGFR via its ubiquitin-interacting motifs, and then recruits downstream ESCRT complexes (ESCRT-1, -II, and -III) to mediate EGFR intraluminal vesicle sorting (Raiborg et al., 2002). It was previously reported that the mutation of 15 lysine residues to arginine in the TKD (15KR mutant) diminished EGFR ubiquitination to a negligible level, and significantly blocked EGFR lysosomal sorting and degradation, suggesting that ubiquitination on some or all of these lysine residues is critical for EGFR lysosomal sorting and degradation (Huang et al., 2007). In addition, EGFR fused to associated molecule with the Src homology 3 domain of signal transducing adaptor molecule (AMSH), a deubiquitinating enzyme that specifically targets lysine 63-linked polyubiquitin chains, cannot be efficiently transported to the MVBs/lysosomes upon EGF engagement, resulting in prolonged EGFR signaling (Huang et al., 2013). Together, these findings demonstrate the essential role of EGFR ubiquitination in its lysosomal sorting and degradation. Casitas B-lineage lymphoma (CBL) is a well-known E3 ubiquitin ligase that mediates EGFR ubiquitination and trafficking (Levkowitz et al., 1998; Grovdal et al., 2004; Stang et al., 2004). Following EGF binding, CBL is recruited to the activated EGFR at the plasma membrane and remains associated with EGFR after receptor internalization to catalyze EGFR ubiquitination for subsequent lysosomal degradation (de Melker et al., 2001; Umebayashi et al., 2008). Despite the important role of CBL in regulating EGFR ubiquitination and lysosomal degradation, the specific CBL-mediated ubiquitin-conjugated lysine residues on EGFR remain unknown. Recently, two other E3 ubiquitin ligases, RNF126 and Rabring7, were shown to associate with EGFR and promote EGFR ubiquitination and degradation upon ligand engagement; but their functions require CBL activation (Smith et al., 2013). Nevertheless, the EGFR^Y1045F^ mutant, which is unable to directly recruit CBL, is still modified by ubiquitination, but to a lesser extent, after EGF stimulation (Levkowitz et al., 1999), which means other E3 ubiquitin ligases also participate in the ubiquitination of EGFR. Therefore, we were interested in investigating if there were additional E3 ubiquitin ligases involved in EGFR ubiquitination and lysosomal degradation.

The zinc and ring finger 1 (ZNRF1) protein, a ring-type E3 ubiquitin ligase, was initially identified as a nerve injury-induced gene (Araki et al., 2001). We previously found that ZNRF1 regulates the Toll-like receptor 4 (TLR4) signaling pathway during inflammation and promotes caveolin-1 (CAV1) ubiquitination and degradation (Lee et al., 2017). CAV1 has been reported to play a role in regulating EGFR trafficking from early to late endosomes (Schmidt-Glenewinkel et al., 2012). Therefore, we hypothesized that ZNRF1 may modulate endosomal trafficking of EGFR and its downstream signaling.

In the present study, we surprisingly found that ZNRF1 regulates EGFR endocytic trafficking and promotes its degradation via receptor ubiquitination. We show that ZNRF1 associates with and ubiquitinates EGFR. Depletion of ZNRF1 in lung or cervical cancer cells results in decreased EGF-induced EGFR ubiquitination and increased accumulation of EGFR in the early endosomes, which eventually impedes EGFR degradation and leads to prolonged activation of AKT and extracellular signal-regulated kinase (ERK) signaling. Our results identify ZNRF1 as a novel regulator of EGFR signaling through regulation of EGFR ubiquitination, sorting, and degradation.

## Results

### ZNRF1 Controls Ligand-Induced EGFR Degradation and Signaling

**FIGURE 1:**
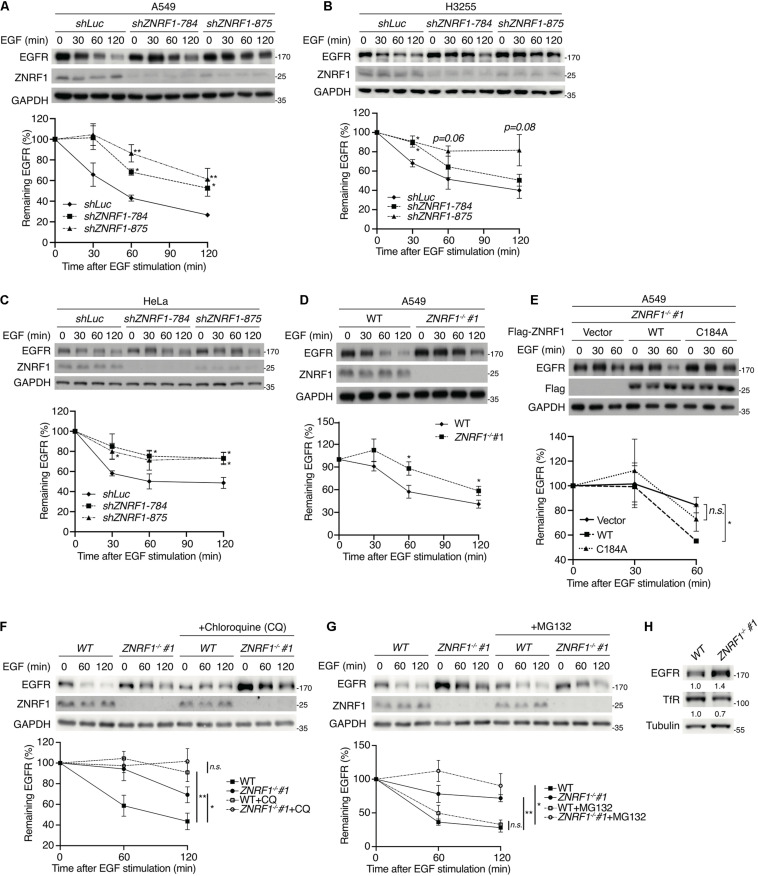
ZNRF1 controls EGFR degradation. **(A–E)** A549 **(A)**, H3255 **(B)**, and HeLa **(C)** cells infected with lentivirus expressing shRNA against luciferase (*shLuc*) or ZNRF1 (*shZNRF1*), wild type or *ZNRF1*^–/–^ A549 cells **(D)**, and *ZNRF1*^–/–^ A549 cells transduced with lentiviruses encoding empty vector, Flag-ZNRF1 (WT), or Flag-ZNRF1 (C184A) **(E)** were serum-starved overnight and treated with 100 ng/mL EGF for the indicated times. **(F,G)** Wild type and *ZNRF1*^–/–^ A549 cells were serum-starved overnight. Cells were then pre-treated with or without 100 μM Chloroquine **(F)** or 10 μM MG132 **(G)** for 1 h and stimulated with 100 ng/mL EGF for the indicated times. Cell lysates were prepared, and the levels of EGFR, ZNRF1 were analyzed by immunoblotting. Quantification of immunoblotting analysis data of three independent experiments are shown in the lower panels. **(H)** Cell lysates from wild type or *ZNRF1*^–/–^ A549 cells were collected and the protein levels of EGFR and TfR were examined by immunoblotting. The intensities of the bands are expressed as fold increases compared to those of control cells (WT) after normalization to tubulin. Results are presented as averages ± SEM. n.s., no significant; *P < 0.05, **P < 0.01 (Student’s *t*-test).

To investigate the role of ZNRF1 in EGFR signaling, we depleted expression of the *ZNRF1* gene in A549 non-small cell lung cancer cells by lentivirus-mediated small hairpin RNA (shRNA) transduction. Four shRNAs against different regions of the human *ZNRF1* gene reduced endogenous ZNRF1 protein expression by >70% as examined by immunoblot analysis (Supplementary Figure 1A). Silencing ZNRF1 in the A549 cells delayed EGFR degradation in response to EGF stimulation, suggesting that ZNRF1 is involved in EGF-induced EGFR degradation (Figure 1A). Loss of ZNRF1 did not affect *EGFR* mRNA expression after EGF stimulation (Supplementary Figure 1B), confirming that its effect on EGFR is at the protein level. Our previous findings (Lee et al., 2017) had suggested that ZNRF1 may modulate EGFR endosomal trafficking through regulation of CAV1 stability. Surprisingly, exogenous overexpression of CAV1 to the level as that in ZNRF1-depeleted cells did not alter ligand-induced EGFR degradation (Supplementary Figure 2), indicating that overexpression of CAV1 is not sufficient to affect EGFR degradation triggered by its ligand. In addition to A549 cells that express endogenous wild type EGFR, similar results were observed in H3255 cells that express a constitutively active mutant EGFR^L858R^ (Janne et al., 2005), indicating that ZNRF1-controlled EGFR degradation is independent of the EGFR mutation status (Figure 1B). Moreover, ZNRF1-mediated EGFR degradation was also observed in HeLa cervical cancer cells (Figure 1C), indicating that ZNRF1 involvement in EGFR degradation is not limited to lung cancer cells.

To exclude the possibility of an off-target effect by shRNAs, we generated *ZNRF1*^–/–^ A549 cells by the CRISPR/Cas9 genomic editing technique. Two *ZNRF1*^–/–^ A549 clones were generated using two different sgRNAs and indel mutations were confirmed by DNA sequencing (Supplementary Figure 1C). Consistent with the results in ZNRF1-silenced cells, EGF-triggered EGFR degradation was delayed in *ZNRF1*^–/–^ A549 cells compared to wild type cells (Figure 1D and Supplementary Figure 1D). To examine whether the E3 ubiquitin ligase activity of ZNRF1 was required for the regulation of EGFR degradation, we examined EGF-induced EGFR degradation in *ZNRF1^–/–^* A549 cells reconstituted with wild type ZNRF1 or an E3 ligase activity inactive mutant of ZNRF1 (C184A) (Araki and Milbrandt, 2003; Lee et al., 2017). As shown in Figure 1E, EGF-induced EGFR degradation was promoted in *ZNRF1^–/–^* cells reconstituted with wild type ZNRF1 but not in cells reconstituted with the ZNRF1 C184A mutant, confirming that the E3 ligase activity of ZNRF1 is required for its modulation of EGFR degradation.

**FIGURE 2:**
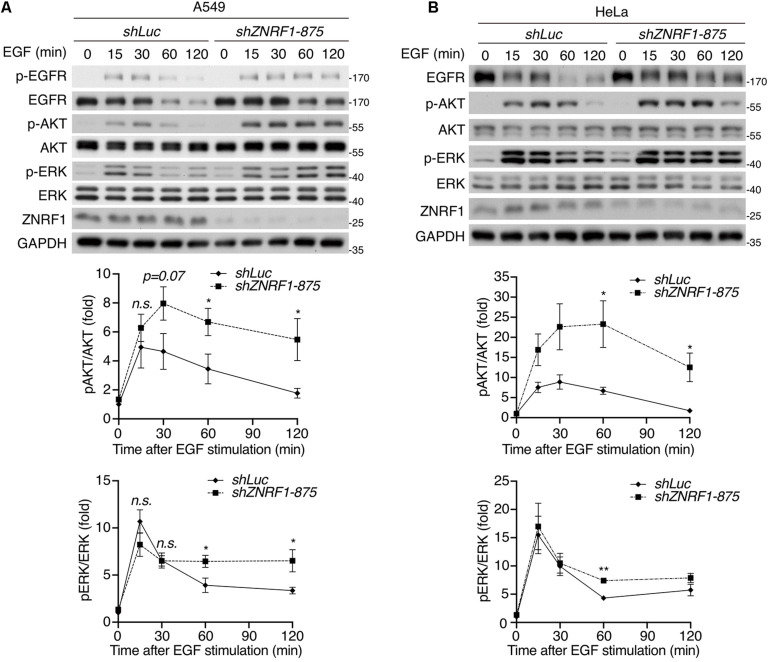
ZNRF1 modulates EGFR signaling. A549 **(A)** and HeLa **(B)** cells infected with lentivirus expressing shRNA against luciferase (*shLuc*) or ZNRF1 (*shZNRF1*) were serum-starved overnight, and then stimulated with 100 ng/mL EGF for the indicated times. Cell lysates were prepared, and the levels of EGFR, ZNRF1, activation of EGFR, AKT, and ERK were analyzed by immunoblotting. Quantification of immunoblotting analysis data of three independent experiments are shown in the lower panels. Results are presented as averages ± SEM. n.s., no significant; *P < 0.05, **P < 0.01 (Student’s *t*-test).

Both the ubiquitin/proteasomal and lysosomal pathways are known to participate in EGFR degradation in response to ligand stimulation (Alexander, 1998; Alwan et al., 2003). To determine the pathway involved in ZNRF1-regulated EGFR degradation, we treated cells with EGF in the presence or absence of the lysosome inhibitor chloroquine or the proteasome inhibitor MG132. Chloroquine treatment significantly attenuated EGFR degradation in control cells, but only had a minor inhibitory effect in ZNRF1-silenced cells (Figure 1F). Conversely, MG132 treatment did not inhibit EGF-induced EGFR degradation in either control or ZNRF1-deleted cells (Figure 1G). These results suggest that ZNRF1-mediated EGFR degradation is dependent on the lysosomal pathway. In line with this notion, deletion of ZNRF1 did not impact protein expression of transferrin receptor (TfR), which is not destined for lysosomal degradation (Figure 1H). We then investigated the impact of ZNRF1 depletion on EGFR signaling and found that loss of ZNRF1 resulted in enhanced and prolonged autophosphorylation of EGFR, and activation of the downstream kinases AKT and ERK in response to EGF stimulation (Figure 2). Taken together, these results indicate that ZNRF1 promotes ligand-triggered EGFR degradation and termination of EGFR signaling via the lysosomal pathway.

### ZNRF1 Promotes EGFR Lysosomal Sorting

**FIGURE 3:**
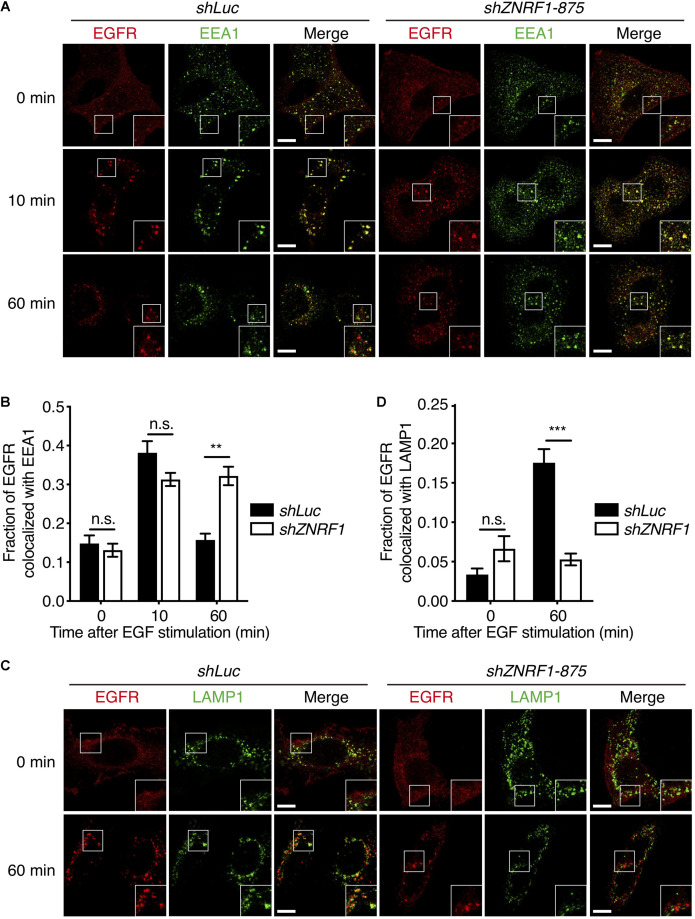
ZNRF1 regulates EGFR trafficking. **(A)** A549 cells expressing *shLuc* or *shZNRF1* shRNA were serum-starved overnight and then stimulated with 100 ng/mL EGF for the indicated times. Cells were co-stained with antibodies against EGFR (red) and EEA1 (green). **(B)** Quantification of the fraction of EGFR co-localized with EEA1 at the indicated times. **(C)** Control and *shZNRF1*-expressing A549 cells pretreated with 100 μM Chloroquine for 1 h and stimulated with 100 ng/mL EGF for 60 min. Cells were stained with antibodies against EGFR (red) and LAMP1 (green). **(D)** Quantification of the fraction of EGFR co-localized with LAMP1 in **(C)**. Data are presented as mean ± SEM, *n* > 500 puncta from 20 cells for each group. Scale bar, 10 μm; n.s., no significant; **P < 0.01, ***P < 0.001. All experiments were repeated two times with similar results.

Endocytic trafficking is one of mechanisms that controls EGFR signaling and degradation (Kirisits et al., 2007; Tomas et al., 2014). To investigate whether ZNRF1 is involved in EGFR trafficking, we first assessed EGFR internalization by tracking the uptake of Alexa Fluor 488-labeled EGF. The amount of internalized EGF was observed to be comparable between control and ZNRF1-depleted cells (Supplementary Figure 3A), indicating that ZNRF1 does not participate in EGFR internalization. We next examined whether ZNRF1 controls EGFR endosomal trafficking, by co-staining EGFR with either the early endosome marker, early endosomal antigen 1 (EEA1), or the late endosome/lysosome marker, lysosomal-associated membrane protein 1 (LAMP1), under EGF stimulation. Co-localization of EGFR and EEA1 was observed in both control and ZNRF1-depleted cells 10 min after EGF stimulation, suggesting that ZNRF1 is not required for EGFR trafficking from the cell surface to the early endosome (Figures 3A,B). However, EGFR-EEA1 co-localization in ZNRF1-depleted cells was significantly higher than in control cells 60 min after EGF stimulation (Figures 3A,B), indicating that loss of ZNRF1 blocked EGFR transport beyond the early endosome. Furthermore, co-localization of EGFR and LAMP1 was significantly reduced in ZNRF1-depleted cells (Figures 3C,D). It was reported that internalized EGFR is recycled back to the cell surface after EGF stimulation (Sorkin et al., 1991). However, we observed no difference in EGFR recycling to the plasma membrane between control and ZNRF1 knockdown cells (Supplementary Figures 3B–D). These data indicate that ZNRF1 regulates EGFR trafficking from early endosomes to late endosomes/lysosomes.

### ZNRF1 Associates With EGFR

**FIGURE 4:**
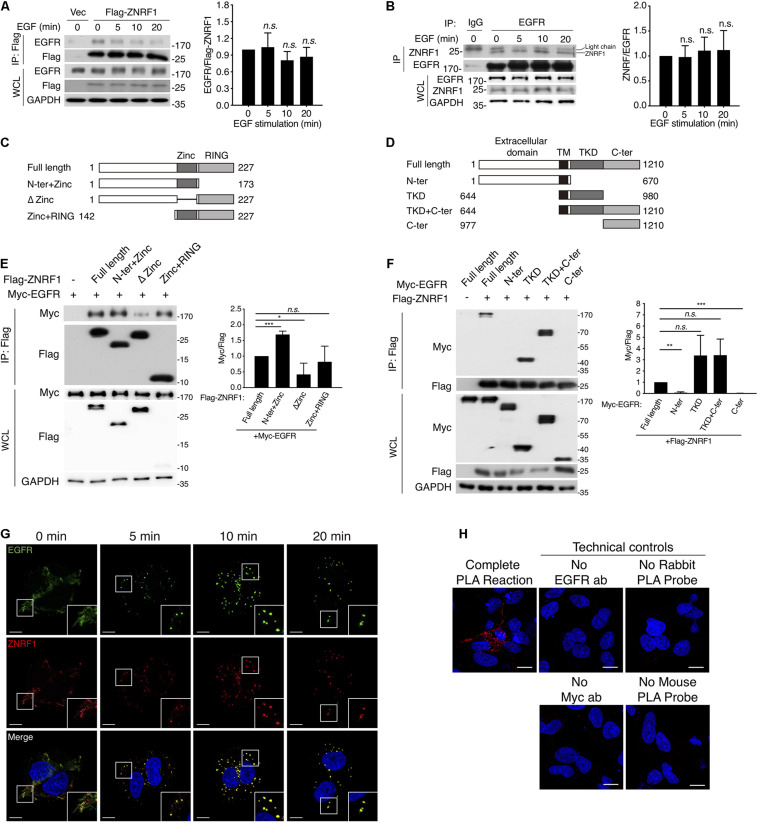
ZNRF1 zinc domain interacts with EGFR TKD. **(A,B)** A549 cells expressing empty vector or Flag-ZNRF1 **(A)** and A549 cells **(B)** were serum-starved overnight and stimulated with 100 ng/mL EGF for the indicated times. EGFR was immunoprecipitated with the indicated antibodies. The immunocomplexes as well as whole cell lysates (WCL) were subjected to immunoblotting with the indicated antibodies. **(C)** Schematic diagram of full-length ZNRF1 and various deletion mutants of ZNRF1 with a C-terminal Flag tag. **(D)** Schematic diagram of full-length and truncated mutants of EGFR with a C-terminal Myc tag. TM, transmembrane domain; TKD, tyrosine kinase domain. **(E)** HEK293T cells were co-transfected with Myc-tagged EGFR and Flag-tagged full-length or truncated forms of ZNRF1 for 48 h, and interactions between EGFR and ZNRF1 were assessed by immunoprecipitation and immunoblotting with the indicated antibodies. **(F)** Flag-tagged ZNRF1 and Myc-tagged full-length or truncated mutants of EGFR were co-expressed in HEK293T cells, and the interactions between EGFR and ZNRF1 were determined by immunoprecipitation and immunoblotting with the indicated antibodies. Quantification of immunoblotting analysis data of three independent experiments are shown in the right panels. Results are presented as averages ± SEM. **(G)** A549 cells were serum-starved overnight and stimulated with 100 ng/mL EGF for the indicated times. Cells were fixed and co-stained with antibodies against ZNRF1 (red) and EGFR (green), and with DAPI (blue), followed by confocal microscopy. **(H)** A549 cells were transfected with Myc-tagged ZNRF1 for 48 h and then subjected to PLA using antibodies against EGFR and Myc. The detected interaction between ZNRF1 and EGFR is represented by red dots, and DAPI-stained cell nuclei are in blue. Scale bar, 10 μm. The experiment was repeated two times with similar results. n.s., no significant; *P < 0.05, **P < 0.01, ***P < 0.001 (Student’s *t*-test).

It is well established that EGF stimulation induces EGFR ubiquitination, which is crucial for receptor sorting to the lysosome for degradation (Huang et al., 2006, 2013). ZNRF1 has been shown to mediate ubiquitination and degradation of AKT and CAV1 (Araki and Milbrandt, 2003; Wakatsuki et al., 2011; Lee et al., 2017), which prompted us to speculate that ZNRF1 might control EGFR trafficking by modulating EGFR ubiquitination. To address this possibility, we first examined whether ZNRF1 associates with EGFR. Reciprocal co-immunoprecipitations revealed an interaction between EGFR and ZNRF1 in A549 cells that transiently overexpressed ZNRF1 (Figure 4A). In addition, an association between endogenous EGFR and ZNRF1 was observed in A549 cells with and without EGF stimulation (Figure 4B). To identify the domains of ZNRF1 and EGFR required for their interaction, we constructed three ZNRF1 domain deletion mutants (Figure 4C) and four truncated forms of EGFR (Figure 4D) for co-immunoprecipitation experiments. In 293T cells, deletion of the ZNRF1 zinc finger domain strongly impeded ZNRF1 binding to EGFR (Figure 4E), indicating that the zinc finger domain mediates the ZNRF1-EGFR interaction. Two truncated forms of EGFR, TKD and TKD plus C-terminal domain, exhibited binding to ZNRF1 that was similar to full-length EGFR, whereas the N-terminal and C-terminal domains lost their association with ZNRF1 (Figure 4F), indicating that the TKD of EGFR is required for its interaction with ZNRF1. These results suggest that the zinc finger domain of ZNRF1 binds to the TKD of EGFR.

We further examined co-localization of ZNRF1 and EGFR in A549 cells by immunofluorescence staining. EGF stimulation rapidly induced EGFR internalization and accumulation in endosomes observed as large puncta in the cytosol. Co-immunofluorescence staining of ZNRF1 and EGFR revealed that ZNRF1 co-localized with these puncta (Figure 4G), consistent with their association revealed by co-immunoprecipitation. To confirm the interaction of ZNRF1 and EGFR *in situ*, we conducted a proximity ligation assay (PLA). Compared to the technical controls that lacked at least one essential component of the system, clear cytosolic fluorescence signals were detected in A549 cells (Figure 4H), demonstrating *in situ* interaction of ZNRF1 and EGFR. Taken together, these results confirm that ZNRF1 associates with EGFR.

### ZNRF1 Mediates EGF-Induced EGFR Ubiquitination for Receptor Recruitment of the ESCRT Machinery

**FIGURE 5:**
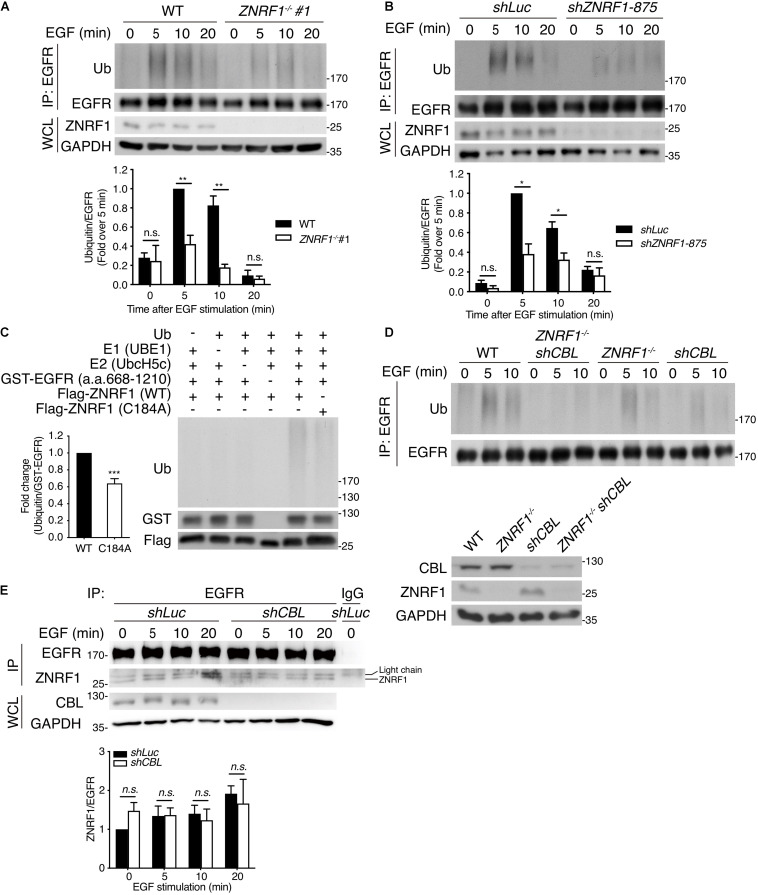
ZNRF1 mediates EGF-induced EGFR ubiquitination. **(A)** Wild type (WT) and *ZNRF1*^–/–^ **(B)** *shLuc* and *shZNRF1*-expressing A549 cells were serum-starved overnight and stimulated with 100 ng/mL EGF for the indicated times. EGFR was immunoprecipitated with anti-EGFR antibody, followed by immunoblotting with anti-ubiquitin and anti-EGFR antibodies. **(C)** *In vitro* ubiquitination assays were conducted with bacterially expressed Flag-tagged WT ZNRF1 or ZNRF1(C184A) mutant together with recombinant ubiquitin, E1, E2 (UbcH5c), and GST-tagged EGFR (a.a. 668–1210) as indicated. The reaction mixtures were subjected to immunoblotting using antibodies against ubiquitin, GST, and Flag antibodies. **(D,E)** Wild type, *ZNRF1*^–/–^, *shCBL*, or *shCBL*-expressing *ZNRF1*^–/–^ A549 cells **(D)**, or A549 cells expressing *shLuc* or *shCBL* shRNA **(E)** were serum-starved overnight and stimulated with 100 ng/mL EGF for the indicated times. EGFR was immunoprecipitated and the immunocomplexes as well as WCL were subjected to immunoblotting with the indicated antibodies. Quantification of immunoblotting analysis data of three independent experiments are shown in the lower panels. Results are presented as averages ± SEM. n.s., no significant; *P < 0.05, **P < 0.01, ***P < 0.001 (Student’s *t*-test).

To determine if ZNRF1 modulates EGFR ubiquitination, we first examined endogenous EGFR ubiquitination following EGF stimulation. EGF induced EGFR ubiquitination within 5–10 min in wild type cells. However, EGF-induced EGFR ubiquitination was markedly diminished in *ZNRF1^–/–^* cells (Figure 5A) and *ZNRF1*-knockdown A549 cells (Figure 5B). We next performed an *in vitro* ubiquitination assay to investigate whether ZNRF1 directly catalyzes EGFR ubiquitination. A recombinant human EGFR peptide (a.a. 668–1210) containing the cytosolic region of EGFR was incubated with a recombinant ZNRF1 protein and other essential components for ubiquitination. Our results show strong poly-ubiquitination of EGFR only in the presence of wild type ZNRF1, but not the ligase inactive C184A mutant (Figure 5C). These data indicate that ZNRF1 directly mediates EGFR ubiquitination in response to EGF stimulation. Given that CBL is a well-known E3 ubiquitin ligase involved in EGFR ubiquitination, endocytic trafficking, and degradation (Levkowitz et al., 1998; de Melker et al., 2001; Duan et al., 2003; Ravid et al., 2004; Umebayashi et al., 2008), we then investigated whether both ZNRF1 and CBL were required for EGFR ubiquitination. We knocked down *CBL* expression by shRNA in wild type or *ZNRF1^–/–^* A549 cells and examined EGFR ubiquitination after EGF stimulation. Loss of either *CBL* or *ZNRF1* reduced EGFR ubiquitination, however EGFR ubiquitination was further decreased to a negligible level in *ZNRF1/CBL* double deficient cells (Figure 5D). Notably, the interaction of EGFR and ZNRF1 was comparable between control and *CBL*-knockdown cells (Figure 5E). Similarly, depletion of ZNRF1 did not affect the association of EGFR and CBL (Supplementary Figure 4). These data indicate that ZNRF1 may function together with CBL to mediate EGFR ubiquitination and degradation.

**FIGURE 6:**
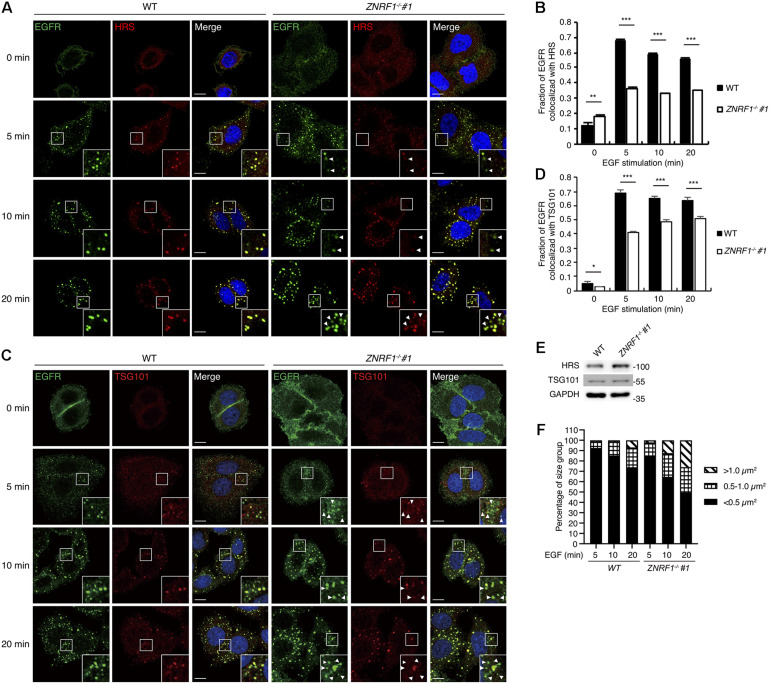
ZNRF1 mediates the recruitment of ESCRT machinery to internalized EGFR. **(A–D)** Wild type or *ZNRF1*^–/–^ A549 cells were serum-starved overnight and stimulated with 100 ng/mL EGF for 0, 5, 10, and 20 min. **(A)** Cells were co-stained with EGFR (Green) and HRS (Red) antibodies. **(B)** Quantification of the fraction of EGFR co-localized with HRS in **(A)**. **(C)** Cells were co-stained with EGFR (Green) and TSG101 (Red) antibodies. **(D)** Quantification of the fraction of EGFR co-localized with TSG101 in **(C)**. **(E)** Cell lysates from wild type and *ZNRF1*^–/–^ A549 cells were prepared and the levels of HRS and TSG101 were analyzed by immunoblotting. **(F)** Quantification of the size distribution of EGFR puncta in **(A)**. White arrows indicate non-colocalized signals. Scale bar, 10 μm. Data are presented as mean ± SEM. *n* > 1000 puncta from at least 50 cells for each group; *P < 0.05; **P < 0.01; ***P < 0.001. All experiments were repeated two to three times with similar results.

Multiple studies show that during receptor endocytosis, ubiquitinated EGFR is recognized by ubiquitin binding domain-containing proteins including HRS and tumor susceptibility gene 101 (TSG101), which are essential components of the ESCRT machinery (Raiborg and Stenmark, 2009; Eden et al., 2012), eventually leading to receptor lysosomal targeting. Therefore, we speculated that reduced EGFR ubiquitination due to ZNRF1 deficiency may decrease its recognition by HRS resulting in decreased receptor lysosomal sorting. To test this hypothesis, we examined the co-localization of EGFR with HRS and TSG101. After ligand stimulation, EGFR showed significantly decreased co-localization with HRS (Figures 6A,B) and TSG101 (Figures 6C,D) in *ZNRF1^–/–^* cells in comparison with wild type cells. Loss of ZNRF1 did not reduce the protein levels of HRS and TSG101, indicating that the decreased EGFR-HRS and EGFR-TSG101 co-localizations in *ZNRF1^–/–^* cells was not the result of protein instability (Figure 6E). In addition, the size of the EGFR puncta was increased under ZNRF1 deficiency, reflecting the accumulation of internalized EGFR in early endosomes (Figure 6F). These results indicate that ZNRF1 modulates EGFR ubiquitination for recruitment of the ESCRT machinery, thereby contributing to receptor lysosomal sorting and degradation.

### ZNRF1 and CBL Catalyze EGFR Ubiquitination on Distinct Lysine Residues Within the TKD of EGFR

**FIGURE 7:**
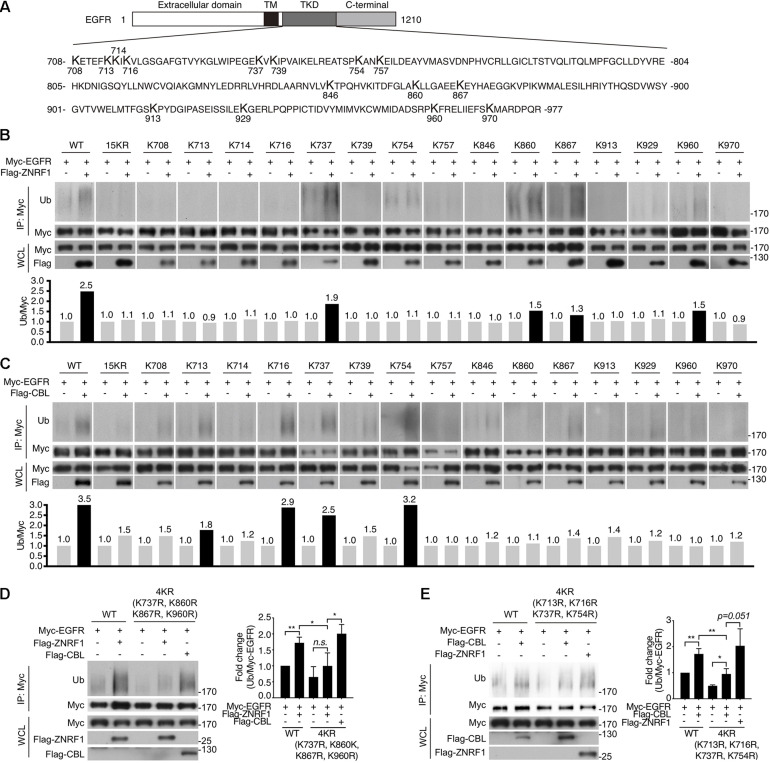
Identification of ubiquitin-modified lysine residues on EGFR mediated by ZNRF1 and CBL in response to EGF. **(A)** Schematic diagram of EGFR TKD sequence and its 15 lysine residues. **(B–E)** HeLa cells were co-transfected with wild type or various EGFR mutants in combination with an empty vector or the E3 ubiquitin ligase ZNRF1 or CBL as indicated for 48 h. Cells were serum-starved and stimulated with 100 ng/mL EGF for 5 min. Ubiquitination of EGFR was examined by immunoprecipitation with Myc-conjugated agarose, followed by immunoblotting with anti-ubiquitin and anti-Myc antibodies. The experiments in **(B,C)** were repeated two times with similar results. Quantification of immunoblotting analysis data of three independent experiments are shown in the left panels of **(D,E)**. Results are presented as averages ± SEM. n.s., no significant; *P < 0.05, **P < 0.01 (Student’s *t*-test).

Previously, analysis of EGF-induced EGFR ubiquitination by mass spectrometry revealed that ubiquitinated lysine residues were located in the TKD of EGFR (Huang et al., 2006). Interestingly, the specific lysine residues for the CBL-mediated polyubiquitination have not been identified. To investigate the differences in EGFR ubiquitination by ZNRF1 and CBL, we sought to identify the acceptor residues for polyubiquitin chains mediated by these two E3 ubiquitin ligases. We co-transfected HEK293T cells with EGFR and ZNRF1 or CBL, and immunoprecipitated EGFR for liquid chromatography-tandem mass spectrometry analysis. In two independent analyses, Lys^716^, Lys^757^, and Lys^860^ of EGFR were identified as acceptor sites for ZNRF1-mediated ubiquitination, whereas Lys^737^ was found to be an acceptor site for CBL-mediated ubiquitination (Supplementary Figure 5 and Table 1). Due to the fact that the combined lysine residues of EGFR identified by mass spectrometry as targets of ZNRF1- and CBL-mediated ubiquitination are fewer than in previous reports (Huang et al., 2007; Tong et al., 2014), an alternative approach was used to systematically assess which EGFR lysine residues were acceptors for the polyubiquitination mediated by ZNRF1 and CBL, respectively. We first constructed an EGFR mutant (15KR), in which all 15 lysine residues in the TKD previously shown to be responsible for ligand-induced ubiquitination (Huang et al., 2007) were replaced by arginine (Figure 7A). We then generated a series of EGFR mutants by reintroducing individual lysine residues into the EGFR (15KR) mutant, and co-expressed these mutants with ZNRF1 or CBL in HeLa cells for immunoprecipitation and ubiquitination analyses. *In vivo* ubiquitination data revealed that ZNRF1 promoted polyubiquitination of wild type EGFR as well as the EGFR mutants K737, K860, K867, and K960 (Figure 7B and Table 1). CBL promoted polyubiquitination of wild type EGFR as well as the EGFR mutants K713, K716, K737, and K754 (Figure 7C and Table 1). To further confirm that these lysine residues were indeed acceptor sites for ZNRF1- and CBL-mediated ubiquitination, we generated two EGFR mutants, EGFR (K737R/K860R/K867R/K960R) and EGFR (K713R/K716R/K737R/K754R), by substituting the indicated lysine residues with arginine. ZNRF1 did not promote ubiquitination of EGFR (K737R/K860R/K867R/K960R), whereas CBL still facilitated its ubiquitination similar to wild type EGFR (Figure 7D). On the other hand, ubiquitination of EGFR (K713R/K716R/K737R/K754R) by CBL was attenuated, while its ubiquitination was still promoted by ZNRF1 as in wild type EGFR (Figure 7E). Together, these results demonstrate that ZNRF1 and CBL catalyze ubiquitination of EGFR at distinct lysine residues.

**TABLE 1:** Summary of ubiquitin-modified lysine residues on EGFR mediated by ZNRF1 and CBL in response to EGF.

Lysine no.	Residues identified by mass spectrometry	Residues identified by *In vivo* ubiquitination assay
708		
713		CBL
714		
716	ZNRF1	CBL
737	CBL	ZNRF1/CBL
739		
754		CBL
757	ZNRF1	
846		
860	ZNRF1	ZNRF1
867		ZNRF1
913		
929		
960		ZNRF1
970		

*HEK293T cells were transfected with Myc-tagged EGFR with empty vector, ZNRF1, or CBL for 48 h. Cell lysates were prepared and subjected to immunoprecipitation with Myc-conjugated agarose, followed by mass spectrometry to identify the ubiquitinated residues on EGFR. The table summarizes the lysine residues identified by mass spectrometry and the *in vivo* ubiquitination assay described in Figure 7. The original contributions presented in the study are publicly available. This data can be found here: [DOI: 10.6019/PXD024279/accession number:PXD024279].*

### ZNRF1 Deficiency Increases Susceptibility to HSV-1 Infection

**FIGURE 8:**
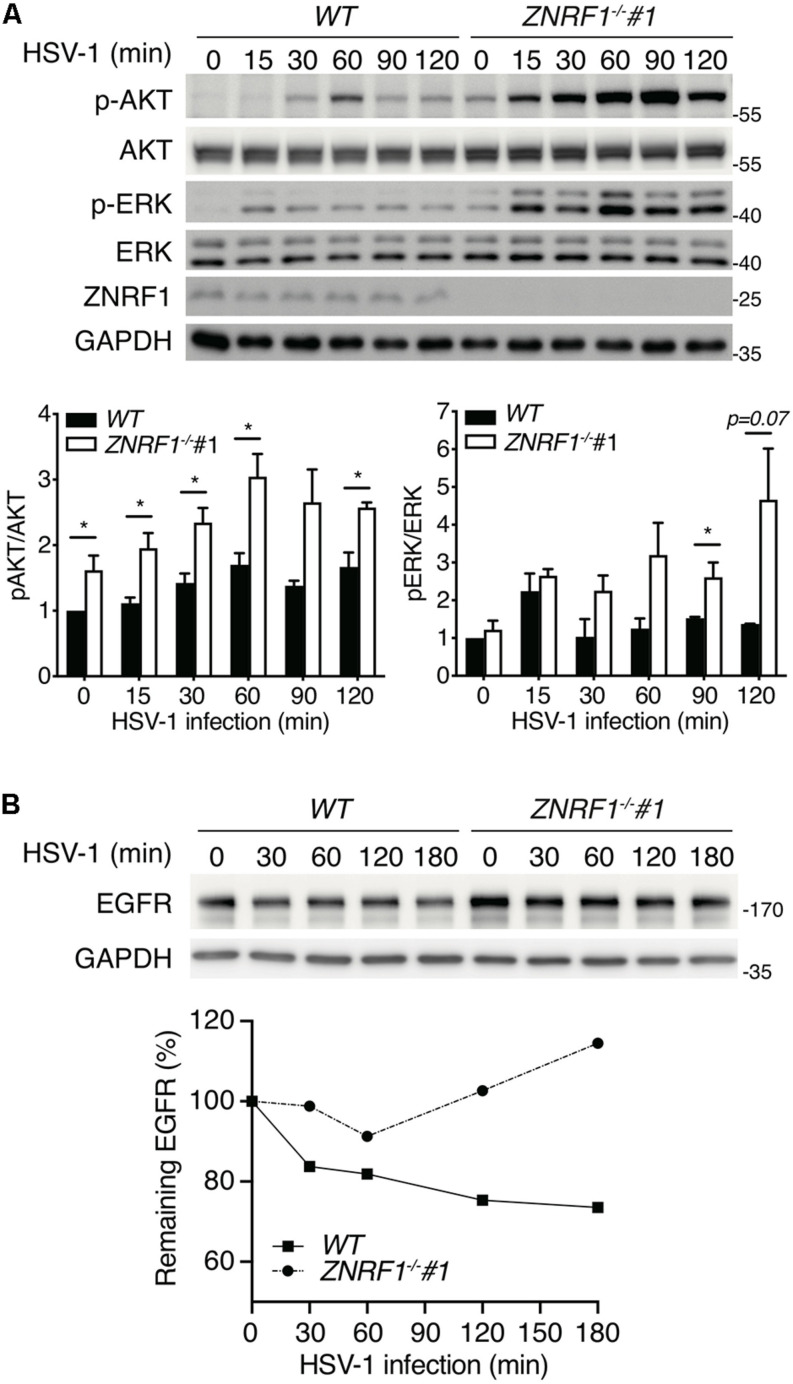
ZNRF1 depletion enhances EGFR signaling after HSV-1 infection. **(A)** Wild type (WT) and *ZNRF1*^–/–^ A549 cells were infected with HSV-1 (MOI = 10) for the indicated times. Activation of AKT and ERK was detected by immunoblotting. Quantification of immunoblotting analysis data of three independent experiments are shown in the lower panel. *P < 0.05 (Student’s *t*-test). **(B)** Wild type and *ZNRF1*^–/–^ A549 cells were serum-starved overnight and infected with HSV-1 (MOI = 15) for the indicated times. The protein levels of EGFR were detected by immunoblotting, quantified, and compared to untreated cells after normalization to GAPDH level. Quantification of EGFR level is shown in the lower panel. The data are representative of three independent experiments.

**FIGURE 9:**
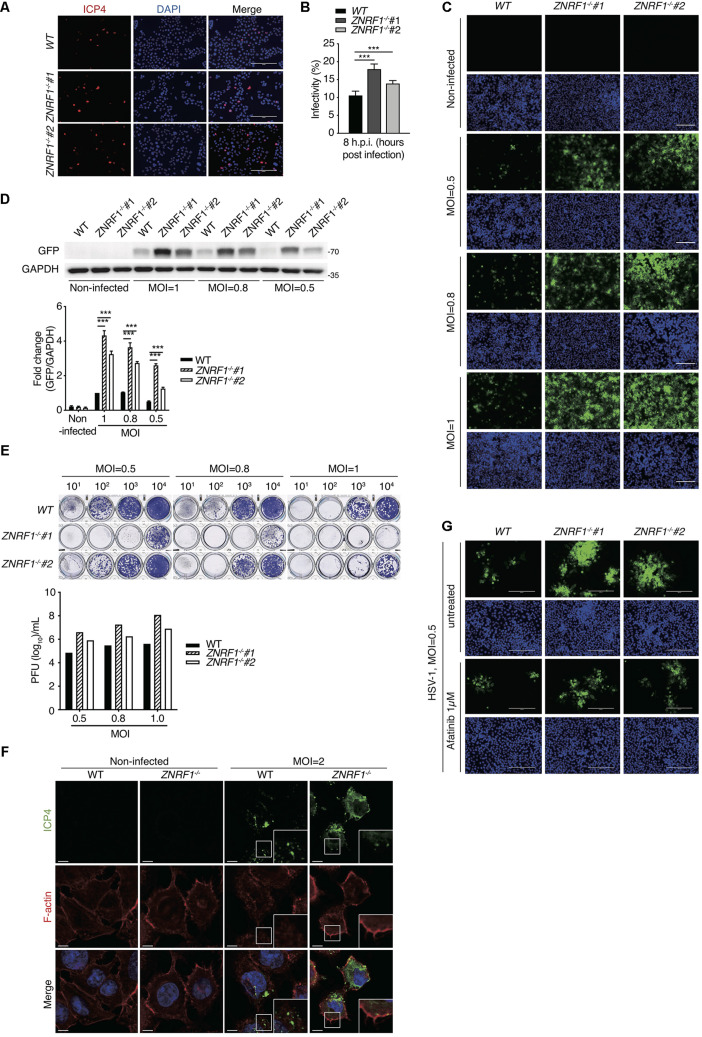
ZNRF1 depletion enhances the entry of HSV-1 into A549 cells. **(A)** Wild type and *ZNRF1*^–/–^ A549 cells were infected with HSV-1 (MOI = 8) for 8 h. Cells were fixed and stained with anti-ICP4 antibody (red) and DAPI (blue). **(B)** HSV-1 infectivity in **(A)** was quantified by the percentage of ICP4-positive cells in five random microscopic fields. Scale bar, 200 μm. ***P < 0.001. **(C–E)** Wild type and *ZNRF1*^–/–^ A549 cells were infected with GFP-expressing HSV-1 (MOI = 0.5, 0.8, or 1.0) for 48 h. **(C)** Cells were collected and analyzed by fluorescence microscopy. **(D)** Cell lysates were collected and subjected to immunoblotting using anti-GFP antibody. Quantification of immunoblotting analysis data of three independent experiments are shown in the lower panel. **(E)** The titers of HSV-1 in culture medium from **(C)** were determined by a plaque assay using Vero cells. **(F)** Wild type and *ZNRF1*^–/–^ A549 cells were infected with HSV-1 (MOI = 2) for 2 h. Cells were co-stained with ICP4 antibody (green) and filamentous actin (F-actin) (red). **(G)** WT and *ZNRF1^–/–^* A549 cells were pretreated with 1 μM Afatinib for 1 h and then infected with GFP-expressing HSV-1 (MOI = 0.5) for 48 h. Cells were collected and analyzed by fluorescence microscopy. Scale bar, 200 μm. Results are presented as averages ± SEM. ***P < 0.001 (Student’s *t*-test). The data are representative of three independent experiments.

HSV-1, a highly prevalent pathogen in the human population, hasdeveloped numerous strategies to boost its capability to infect abroad range of host cells, including multiple entry modes and alternative receptors (Karasneh and Shukla, 2011). One of its entry strategies is to induce actin cytoskeleton remodeling via activation of EGFR signaling in host cells to facilitate viral entry (Zheng et al., 2014). We assessed whether dysregulated EGFR signaling caused by ZNRF1 deficiency enhances HSV-1 entry during early infection. HSV-1 infection induced a rapid activation of AKT and ERK in A549 cells, and the activity of both kinases was enhanced and prolonged in *ZNRF1^–/–^* cells compared to wild type cells (Figure 8A). Not surprisingly, EGFR degradation in response to HSV-1 infection was delayed in ZNRF1-deficient cells (Figure 8B). Next, we examined the expression of infected cell polypeptide 4 (ICP4), a viral immediate early gene required for transcription of early and late viral genes in HSV-1 infected cells (Smith et al., 1993; Lester and DeLuca, 2011). At 8 h post infection, ICP4 signals were significantly increased in *ZNRF1^–/–^* cells, indicating that loss of ZNRF1 increased HSV-1 infectivity (Figures 9A,B). Furthermore, a higher viral load was detected in *ZNRF1^–/–^* cells infected with HSV-1 expressing GFP at 48 h post infection in comparison to wild type cells (Figures 9C–E). Consistent with prolonged EGFR activation, actin cytoskeleton rearrangement was promoted in HSV-1-infected *ZNRF1*^–/–^ cells (Figure 9F). Inhibition of EGFR kinase activity by EGFR inhibitor Afatinib reduced HSV-1-GFP signals in *ZNRF1^–/–^* cells to the level that is similar to WT cells (Figure 9G), confirming that increased HSV-1 infectivity in *ZNRF1^–/–^* cells required EGFR activation. Taken together, these results suggest that ZNRF1 controls EGFR degradation and activation of its downstream signaling upon HSV-1 infection, which may contribute to host defense by constraining HSV-1 infection.

## Discussion

Epidermal growth factor receptor signaling is essential for numerous cellular functions, including cell proliferation, survival, differentiation, and the immune response (Schlessinger, 2002; Yamashita et al., 2012; Chattopadhyay et al., 2015). Although EGFR signaling is activated primarily at the plasma membrane, activated EGFR continues to deliver downstream signals from the early endosomes (Wang et al., 2002; Brankatschk et al., 2012; Sousa et al., 2012). Trafficking of EGFR to MVBs/lysosomes for the degradative pathway is critical for termination of EGFR signaling. Ubiquitination of EGFR is essential for EGFR sorting from the endosomes to MVBs/lysosomes. In this study, in addition to CBL, we identified an E3 ubiquitin ligase ZNRF1 that modulates EGFR ubiquitination, leading to EGFR trafficking to the MVBs/lysosomes for degradation. ZNRF1 deficiency resulted in decreased ligand-induced EGFR ubiquitination, thereby increasing endosomal accumulation of EGFR, leading to prolonged activation of the downstream pathways and enhanced HSV-1 infectivity. Our findings reveal ZNRF1 as a novel regulator of the EGFR signaling pathway that functions together with CBL by controlling EGFR ubiquitination, endosomal trafficking, and degradation.

ZNRF1 was originally identified in injured neurons (Araki et al., 2001) and later shown to exhibit E3 ubiquitin ligase activity (Araki and Milbrandt, 2003). Previous studies have demonstrated that ZNRF1 plays a critical role in Schwann cell differentiation and Wallerian degeneration by controlling the degradation of glutamine synthetase and AKT via the ubiquitin–proteasome pathway, respectively (Saitoh and Araki, 2010; Wakatsuki et al., 2011). Recently, we found that ZNRF1 modulates the Toll-like receptor 4-triggered immune response by targeting CAV1 ubiquitination and degradation (Lee et al., 2017). The expression of CAV1 protein was also elevated in ZNRF1-depleted A549 cells, but increased CAV1 was not sufficient to influence EGFR degradation triggered by its ligand. Nevertheless, we cannot rule out the possibility that defective CAV1 ubiquitination and degradation contributes to ligand-induced EGFR trafficking and degradation in ZNRF1 deficient cells. Our current findings unveil a novel function of ZNRF1 by modulating ligand-induced EGFR ubiquitination and degradation through the lysosomal degradation system.

Ubiquitination of EGFR is rapidly induced upon EGF stimulation. Accumulating data show that while EGFR ubiquitination is dispensable for its internalization, it is crucial for receptor sorting (Huang et al., 2007, 2013). Prior to this study, CBL was an key E3 ubiquitin ligase involved in mediating ligand-induced EGFR ubiquitination and lysosomal degradation, although two other E3 ubiquitin ligases were recently reported to participate in this events and act downstream of CBL (Smith et al., 2013). In addition, despite the discovery of CBL decades ago, its ubiquitin acceptor residues on EGFR have not been identified. Our findings indicate that ZNRF1 in combination with CBL regulate EGFR ubiquitination and lysosomal sorting. Deletion of both CBL and ZNRF1 diminished EGF-induced EGFR ubiquitination to a negligible level.

To identify the ubiquitination sites on EGFR mediated by ZNRF1 and CBL, we first immunoprecipitated receptors from HEK293T cells co-expressing EGFR and CBL or ZNRF1 for LC-MS analysis, which can assess EGFR ubiquitination on a proteome-wide level. We only mapped four ubiquitinated lysine residues on EGFR; Lys716, Lys757, and Lys 860 modulated by ZNRF1, and Lys737 modulated by CBL. This number is fewer than previous reported number of ubiquitinated lysine residues on EGFR (Huang et al., 2007; Tong et al., 2014). It is possible that low-abundance ubiquitinated EGFR peptides were missing during LC-MS analysis. We therefore performed an alternative approach, which was used systematic lysine-to-arginine mutagenesis in combination with *in vivo* ubiquitination assay. This approach allowed us to systematically assess which lysine residues in EGFR kinase domain were acceptors for the polyubiquitination mediated by ZNRF1 or CBL. By using this approach, we identified seven EGF-activated ubiquitin-conjugated lysine residues on EGFR: Lys737, Lys860, Lys867, and Lys960 mediated by ZNRF1, and Lys713, Lys716, Lys737, and Lys754 mediated by CBL. Our results indicate that ZNRF1 and CBL conjugate ubiquitin moieties to distinct lysine residues on EGFR. Interestingly, Lys716, Lys737, Lys754, and Lys867 were previously identified as ubiquitin acceptor sites on ligand-activated EGFR by mass spectrometry (Huang et al., 2006). Our findings are in agreement with the idea that the extent of ubiquitination in the EGFR kinase domain is correlated with the efficiency of EGFR lysosomal sorting and degradation (Huang et al., 2007). Nevertheless, based on a previous study (Huang et al., 2007), we only assessed the possible acceptor sites within the 15 lysine residues in the EGFR TKD for ZNRF1- and CBL-mediated ubiquitination in this study. Therefore, we cannot rule out the possibility that ZNRF1 and CBL may catalyze ubiquitination on additional lysine residues that are not located in TKD of EGFR. Moreover, it remains to be determined whether additional E3 ubiquitin ligases besides ZNRF1 and CBL participate in ligand-activated EGFR ubiquitination and subsequent lysosomal degradation.

Following receptor internalization into the early endosomes, the ubiquitinated receptor is recognized by the ESCRT machinery, allowing the cargo receptor to be destined for lysosomal degradation (Henne et al., 2011). The ESCRT-0 complex contains two ubiquitin-binding proteins, HRS and signal transducing adaptor molecule, which interact with the ubiquitinated receptor and initiate its intraluminal sorting. HRS is recruited to the endosome membrane through its binding to ubiquitinated proteins via its ubiquitin-interacting motif (Raiborg et al., 2002; Hirano et al., 2006). Our data show that a lack of ZNRF1-mediated EGFR ubiquitination decreased activated EGFR co-localization with HRS, suggesting reduced recruitment of HRS to ubiquitinated EGFR in the early endosomes. In addition, perturbations of the interaction between HRS and TSG101, a component of the ESCRT-I complex, block lysosomal trafficking of EGFR, leading to an accumulation of EGFR in the early endosomes (Lu et al., 2003). Moreover, our findings are consistent with the suggestion that multiple ubiquitin moieties on EGFR are required for efficient binding to the ESCRT machinery (Huang et al., 2006; Umebayashi et al., 2008; Raiborg and Stenmark, 2009). Many studies have demonstrated that when EGFR fails to bind to HRS, the receptor becomes trapped in the lining membrane of the endosomes, reflected by enlarged endosomes without intraluminal vesicles. In agreement with published data, we observed enlargement of EGFR-positive endosomes following ligand stimulation in ZNRF1-depleted cells. Taken together these results suggest that ZNRF1 mediates ligand-induced EGFR ubiquitination, which is required for a sufficient sorting signal for the recruitment of HRS.

Casitas B-lineage lymphoma is known to be recruited to activated EGFR at the plasma membrane upon ligand stimulation, and the carboxy-terminal domain of EGFR is critical for this interaction (Levkowitz et al., 1999; Waterman et al., 2002). We showed that ZNRF1 associates with the TKD of EGFR, indicating that ZNRF1 and CBL bind different regions of EGFR. Notably, when and how the ligase activity of ZNRF1 is induced upon EGF treatment remains unclear. A recent study in neuronal cells showed that under oxidative stress, ZNRF1 is phosphorylated at its Tyr^103^ residue by EGFR, which is critical for its E3 ligase activity (Wakatsuki et al., 2015). Hence, it is possible that ZNRF1 is phosphorylated by activated EGFR upon EGF stimulation, thereby allowing it to mediate EGFR ubiquitination, trafficking, and degradation. Further studies are required to verify this possibility.

Uncontrolled EGFR signaling is associated with numerous diseases, including cancer (Roepstorff et al., 2008; Tomas et al., 2014). In addition, EGFR activation is crucial for viral entry during HSV-1 infection, which causes a wide range of symptoms from skin lesions to deadly encephalitis. Considering the key role of EGFR ubiquitination and endosomal trafficking in terminating EGFR signaling, ZNRF1 likely acts in concert with CBL to regulate EGFR-mediated cellular functions. Further investigations of ZNRF1 function in the spatiotemporal regulation of EGFR trafficking and signaling may help in the design of novel therapeutic interventions for the treatment of diseases caused by uncontrolled EGFR signaling. Furthermore, HSV-1 has emerged as a promising oncolytic virus for the killing of cancer (Sokolowski et al., 2015), but infectivity efficiency of modified oncolytic HSV-1 is the major limiting factor in the application of oncolytic virus therapy for cancer treatment (Sanchala et al., 2017). In combination with ZNRF1 interference, which significant increases the infectivity of HSV-1, it may increase the efficacy of oncolytic virus therapy for the elimination of cancer.

## Materials and Methods

### Generation of ZNRF1 Knockdown and Knockout Cells

ZNRF1 knockdown and knockout cells were generated using lentiviruses-mediated shRNAs transduction and CRISPR/Cas9/sgRNA system, respectively. Lentivirus production and transduction were performed following the instructions provided by the National RNAi Core Facility Platform at the Institute of Molecular Biology/Genomic Research Center, Academia Sinica, Taiwan.

### Cell Culture and Plasmids

Non-small cell lung cancer A549 and H3255 cells were maintained in RPMI-1640 (Gibco, Carlsbad, CA, United States) supplemented with 10% (vol/vol) heat-inactivated fetal bovine serum (FBS) (Gibco) at 37°C with 5% CO_2_. Human embryonic kidney 293T (HEK293T) and HeLa cells were cultured in high glucose DMEM (Gibco) containing 10% FBS. Chinese hamster ovary (CHO) cells were maintained in Ham’s F-12K (Kaighn’s Modification) (Gibco) containing 10% FBS. Plasmids encoding full-length ZNRF1 cDNA and deletion mutants were described previously (Lee et al., 2017). Flag-tagged ZNRF1 was cloned into the pcDNA3-myc vector (Addgene, Cambridge, MA, United States). The Myc-tagged EGFR expression construct was kindly provided by Dr. Ming-Shiue Lee (Institute of Biochemistry and Molecular Biology, National Taiwan University, Taiwan). The truncated forms of EGFR, including N-terminal, TKD, TKD plus C-terminal, and C-terminal, were generated by PCR using full-length EGFR cDNA as a template and cloned into the pcDNA3-myc vector (Addgene). The EGFR lysine-to-arginine substitution mutants were generated by PCR using the Q5^®^ site-directed mutagenesis Kit (New England Biolabs, Ipswich, MA, United States) following the manufacturer’s instructions.

### Reagents and Antibodies

The recombinant human EGF protein and Alexa Fluor 488-conjugated EGF protein were purchased from Thermo Fisher Scientific (Boston, MA, United States). Antibodies against pEGFR Ser^1068^ (#3777), EGFR (#4267), pAKT Ser^473^ (#4060), AKT (#4691), pERK1/2^Thr202/Tyr204^ (#9101), ERK1/2 (#9102), CBL (#8447), HRS (#15087), V5-tag (#13202), and Myc-tag (#2278) were obtained from Cell Signaling Technology (Danvers, MA, United States). GAPDH (GTX627408) and TSG101 (GTX70255) antibodies were purchased from GeneTex (Irvine, CA, United States). Antibodies against Ubiquitin (P4D1) (sc-8017), GFP (sc-8334), and LAMP1 (sc-20011) were obtained from Santa Cruz Biotechnology (Santa Cruz, CA, United States). The anti-FLAG M2 affinity gel (A2220) and anti-c-Myc (A7470) agarose beads were purchased from Sigma-Aldrich (St Louis, MO, United States). EEA1 (#610456) and GST (#554805) antibodies was purchased from BD Bioscience (San Jose, CA, United States). ICP4 (ab6514) antibody was obtained from Abcam (Cambridge, United Kingdom). Rhodamine phalloidin (against F-actin) was purchased from Thermo Fisher Scientific (Boston, MA, United States). The anti-transferrin receptor monoclonal antibody (H68.4) was a kind gift from Dr. Ya-Wen Liu (Institute of Molecular Medicine, National Taiwan University, Taiwan). Antibodies against Flag were generated by Dr. Sheng-Chung Lee as described previously (Yang et al., 2013). The generation of the ZNRF1 antibody was described previously (Lee et al., 2017). EGFR kinase inhibitor Afatinib (ab142018) was purchased from Abcam (Cambridge, United Kingdom).

### shRNA-Mediated Gene Silencing and Lentiviral Infection

Generation of replication-defective lentiviruses encoding shRNAs and lentiviral transduction were performed following the instructions provided by the National RNAi Core Facility Platform at the Institute of Molecular Biology/Genomic Research Center, Academia Sinica, Taiwan. In brief, pLKO-shRNA constructs and the packaging plasmids pMD.G and pCMVR8.91 were transfected into HEK293T cells using Turbofect (Fermentas, Schwerte, Germany) according to the manufacturer’s instructions. The lentivirus-containing culture medium was collected 48 and 72 h after transfection. A549 or H3255 cells were infected with lentiviruses in the presence of 8 μg/mL polybrene (Sigma-Aldrich, St. Louis, MO, United States) for 24 h, and then selected in medium containing 2 μg/mL puromycin (Gold Biotechnology, St Louis, MO, United States) until uninfected cells were completely killed. The shRNA target sequences were 5′-CCTCCCTGAGGACACCAAATT-3′ for sh*ZNRF1*-298, 5′-TAT GCCCATGGCAATGGTTAC-3′ for sh*ZNRF1*-362, 5′-CAGCT CGCATAGTGGTTTCAA-3′ for sh*ZNRF1*-784, 5′-ACAACGAT GATGTGCTGACTA-3′ for sh*ZNRF1*-875, 5′-GCCGATGTG AAATTAAAGGTA-3′ for sh*CBL*-694, and 5′-CCAGTGA GTTGGGAGTTATTA-3′ for sh*CBL*-695.

### Generation of ZNRF1 Knockout Cells Using the CRISPR/Cas9 System

HEK293T cells were transfected with lentiviral packaging plasmids pMD.G and pCMVR8.91, and CRISPR/sgRNA/puro expression plasmids encoding sgRNA sequence targeting exon 1 of human *ZNRF1.* Then the culture medium containing lentiviruses was collected and used to infect A549 cells for 24 h followed by puromycin selection. The sgRNA target sequences were 5′-GATTTCGGGCACTACCGGAC-3′ for sgRNA #1 and 5′-GCATTTCGGGCACTACCGGA-3′ for sgRNA #2. To verify gene editing in single cell clones, genomic DNA was purified and subjected to PCR and DNA sequencing. The primers used for PCR were: forward primer 5′-TTGACTCCCTCCCCCTTTATGCTCG-3′ and reverse primer 5′-ATAGGTGGAGTCGGACGCAGACCCT-3′ for clones from sgRNA #1, and forward primer 5′-TTGACTCCCTCCCCCTTTATGCTCG-3′ and reverse primer 5′-ATAGGTGGAGTCGGACGCAGACCCT-3′ for clones from sgRNA #2.

### RNA Isolation and Quantitative RT-PCR (RT-qPCR)

Total cellular RNA was purified using TRIzol RNA Isolation Reagent (Thermo Fisher Scientific) according to the manufacturer’s instructions. One μg of total RNA was then reverse transcribed to cDNA using the ReverAid H Minus First Strand cDNA Synthesis kit (Thermo Fisher Scientific) following the manufacturer’s instructions. 1/20th volume of the cDNA was mixed with SYBR Green PCR Master Mix (Thermo Fisher Scientific) to analyze the amount of specific mRNA. The primer sequences used for RT-qPCR were: Cyclophilin A (forward 5′-AGGTCCCAAAGACAGCAGA-3′ and reverse 5′-TGTGAAGTCACCACCCTGA-3′), and EGFR (forward 5′-ACTCATGCTCTACAACCC-3′ and reverse 5′-CCAATACCTATTCCGTTACAC-3′). The relative EGFR mRNA expression was obtained by normalizing its qPCR value to the level of Cyclophilin A mRNA.

### Immunoblotting

Cells were lysed in ice-cold lysis buffer containing 50 mM Tris-HCl pH 7.5, 150 mM NaCl, 2 mM EDTA, 1% Triton X-100, 0.5% NP-40, 10% Glycerol, protease inhibitors Aprotinin, Benzamidine, Pepstatin A, and Leupeptin (Sigma-Aldrich) and phosphatase inhibitors, sodium orthovanadate and *p*-nitrophenyl phosphate (pNPP) (Sigma-Aldrich). Cell extracts were collected and protein concentrations quantified using the Bio-Rad Protein Assay (Bio-Rad, Hercules, CA, United States). Cell lysates were resolved by SDS-PAGE and transferred to polyvinylidene fluoride (PVDF) membranes (Millipore, Billerica, MA, United States). The membranes were blocked with 10% non-fat milk in TBST (50 mM Tris-HCl pH 7.6, 150 mM NaCl, 0.05% Tween-20), Blocking One (Nacalai Trsque, Nakagyo-ku Kyoto, Japan), or 5% BSA (for phosphorylated protein) for 30 min at 25°C, and then incubated with the indicated primary antibody overnight at 4°C, followed by a horseradish peroxidase-conjugated secondary antibody (Jackson ImmunoResearch, West Grove, PA, United States) for 1 h at 25°C. Immunoreactive signals were detected using Luminata Western Chemiluminescent HRP substrates (Millipore) according to the manufacturer’s instructions.

### Immunoprecipitation

Cells were lysed in ice-cold lysis buffer containing 150 mM NaCl, 50 mM Tris-HCl pH 7.5, 2 mM EDTA, 1% Triton X-100, 0.5% NP-40, 10% Glycerol, protease inhibitors and phosphatase inhibitors. For immunoprecipitation of ubiquitin-modified proteins, 20 mM *N*-ethylmaleimide (Sigma-Aldrich) was added to the lysis buffer. Cell extracts were incubated with anti-FLAG or c-Myc antibody-conjugated agarose beads at 4°C for 1.5 h or the indicated primary antibody overnight at 4°C followed by a 2 h incubation with Protein A Sepharose CL-4B (GE Healthcare, Piscataway, NJ, United States). The immunocomplexes were then subjected to SDS-PAGE followed by immunoblotting.

### Immunofluorescence

Cells were seeded on coverslips and cultured overnight before treatment. Cells were then fixed with 4% paraformaldehyde (PFA) (Electron Microscopy Sciences, Hatfield, PA, United States) in phosphate-buffered saline (PBS) (Gibco) pH 7.4 at 37°C for 15 min and permeabilized with 0.25% Triton X-100 in PBS at room temperature for 15 min, followed by blocking with 1% BSA in PBST (0.25% Triton X-100 in PBS) at 25°C for 30 min. The coverslips were then incubated with primary antibody overnight at 4°C, washed with PBS, and stained with a fluorescent-conjugated secondary antibody (Jackson ImmunoResearch) at 25°C for 1 h. After extensive washings with PBS, the coverslips were mounted with DAPI Fluoromount-G (SouthernBiotech, Birmingham, AL, United States) to counterstain cell nuclei. Images were captured using a Zeiss LSM 700 Confocal microscope and the co-localization of EGFR with EEA1 and LAMP1 was analyzed using the ZEN imaging software (Zeiss, Oberkochen, Germany). The co-localization of EGFR with ESCRT complex protein (HRS and TSG101) was analyzed using Volocity 3D imaging software (PerkinElmer, Waltham, MA, United States). Briefly, the images were subtracted from background and then segmented using the minimal intensity of each individual organelle marker-labeled vesicles as the low threshold. The integrated voxel intensity of EGFR in the segmented image was considered as co-localization of EGFR with each individual organelle marker-labeled vesicles. The extent of co-localization was determined as the percentage of integrated EGFR fluorescence of the segmented image to the total fluorescence of the same fluorochromes. To quantify the size of EGFR puncta, the intensities and areas of EGFR signals were acquired and analyzed by Metamorph software (Molecular Devices, San Jose, CA, United States).

### Proximity Ligation Assay (PLA)

Proximity ligation assays were performed by Duolink *In Situ*-Fluorescence Detection Reagent Red (Sigma-Aldrich) according to the manufacturer’s instructions. In brief, cells were seeded on coverslips and cultured overnight followed by transfection of Myc-EGFR expression plasmid for 48 h. Cells were then fixed, permeabilized, and incubated with the primary antibody overnight at 4°C. On the following day, the cells were washed with PBS and incubated with PLA probes for 1 h at 37°C. The cells were then washed twice with buffer A (Sigma-Aldrich), incubated with ligation mixture for 1 h at 37°C, washed twice with buffer A, and incubated in amplification mixture (Sigma-Aldrich) for 100 min at 37°C. After three washes with buffer B (Sigma-Aldrich), the coverslips were mounted with DAPI Fluoromount-G (SouthernBiotech). Images were captured using a Zeiss LSM 700 Confocal microscope (Zeiss, Oberkochen, Germany).

### EGFR Internalization and EGFR Recycling Assays

For the EGFR internalization assay, cells were serum starved overnight, stimulated with 2 μg/mL Alex Fluor 488-conjugated EGF (Thermo Fisher Scientific) for the indicated times at 37°C, and placed on ice to stop internalization. After three washings with ice-cold PBS, cells were subjected to an acid wash (0.2 M acetic and 0.5 M NaCl, pH 2.8) for 5 min at 4°C. Cells were then detached from the culture dishes, washed with PBS, and re-suspended in PBS containing 2% FBS and 0.01% sodium azide, followed by fixation with 4% PFA in PBS for 20 min. Fixed cells were analyzed by a BD LSR II flow cytometer (BD Biosciences, San Jose, CA, United States). For the EGFR recycling assay, cells were serum starved overnight, and pretreated with 10 μg/mL cycloheximide for 1 h. All the following steps were performed in the presence of cycloheximide to inhibit new synthesis of EGFR. To obtain the total amount of initial internalized EGFR, cells were stimulated with 2 μg/mL of AF488-conjugated EGF for 15 min at 37°C, and then washed and fixed with 4% PFA in PBS. To obtain the amount of recycled EGFR, cells were first stimulated with 1 ng/mL or 100 ng/mL of non-labeled EGF (Peprotech, Rocky Hill, NJ, United States) for 15 min at 37°C, rinsed three times with PBS, and chased for 1 or 2 h to allow EGFR recycling. Cells were then treated with AF488-conjugated EGF for 15 min, washed, fixed, and analyzed by flow cytometry to quantify the internalized EGF uptake by recycled EGFR. The ratio of recycled EGFR was determined by the total amount of recycled EGFR relative to the total amount of initial internalized EGFR.

### Identification of Ubiquitination Sites by Liquid Chromatography (Nanoflow-LC-MS/MS)

To identify the ubiquitination sites of EGFR mediated by ZNRF1 and CBL, EGFR was isolated by SDS-PAGE followed by in-gel enzymatic digestion with a mixture of trypsin and chymotrypsin. The digested peptides were analyzed by nanoflow LC-MS/MS on an LTQ-FT hybrid mass spectrometer (Thermo Fisher Scientific) equipped with a nano-electrospray ion source (New Objective, Inc., Woburn, MA, United States) in positive ion mode. The liquid chromatography system used was the Agilent 1100 HPLC with the Famos Autosampler (LC Packings, Amsterdam, Netherlands). The digested peptide samples were subjected to nanoflow-LC-MS/MS as described previously (Chang et al., 2019). All experimental RAW files were converted to MGF format by MSConvert (ProteoWizard ver. 3.0.9134) (Chambers et al., 2012) and then submitted for MS/MS ion search on Mascot (ver. 2.3) (MatrixScience, Boston, MA, United States). The protein sequences of Homo sapiens from UniprotKB^1^ were used for MS/MS data analysis. The search parameters of error tolerance of precursor ions and the MS/MS fragment ions in spectra were 10 ppm and 0.6 Da, respectively. The variable post-translational modifications of search parameters in Mascot include ubiquitination of lysine (GlyGly), carbamidomethylation of cysteine, the oxidation of methionine, and phosphorylation of serine/threonine/tyrosine.

### Quantification of HSV-1 Infectivity

For determination of HSV-1 entry, cells were seeded on coverslips and cultured in RPMI-1640 containing 10% FBS overnight. Cells were infected with HSV-1 at 4°C for 1 h, and then washed three times with PBS. After a 8-h incubation at 37°C, cells were fixed and subjected to immunofluorescence with the anti-ICP4 antibody. To detect HSV-1 infection, cells were incubated with GFP-expressing HSV-1 (Elliott and O’Hare, 1999) at 4°C for 1 h to allow adsorption, washed three times with PBS, and incubated at 37°C for 48 h. Infected cells were imaged by fluorescence microscopy, and viral titers in culture medium were determined by a plaque assay on Vero cells. Cell lysates were prepared and subjected to immunoblotting using anti-GFP antibody.

### Statistical Analysis

Results were presented as mean ± SEM. Significant differences between two groups were assessed by the Student’s *t*-test. *P*-values < 0.05 were considered statistically significant.

## Data Availability Statement

The datasets presented in this study can be found in online repositories. The names of the repository/repositories and accession number(s) can be found below: ProteomeXchange, PXD024279.

## Author Contributions

C-CC, C-HS, and L-CH designed the research. C-CC, C-HS, T-YL, J-EH, Y-SL, H-YL, Y-KC, and I-LH performed the experiments. P-HH performed the mass spectrometry analysis. T-HC generated the antibody. C-HS, C-CC, C-YL, and L-CH analyzed the data and wrote the manuscript. All authors contributed to the article and approved the submitted version.

## Conflict of Interest

The authors declare that the research was conducted in the absence of any commercial or financial relationships that could be construed as a potential conflict of interest.

## Footnotes

1 https://www.uniprot.org

## References

[B1] Alexander A. (1998). Endocytosis and intracellular sorting of receptor tyrosine kinases. *Front. Biosci.* 3 d729–38. 10.1101/cshperspect.a017459 9671598

[B2] Alwan H. A. van Zoelen E. J. van Leeuwen J. E. (2003). Ligand-induced lysosomal epidermal growth factor receptor (EGFR) degradation is preceded by proteasome-dependent EGFR de-ubiquitination. *J. Biol. Chem.* 278 35781–35790. 10.1074/jbc.m301326200 12829707

[B3] Araki T. Milbrandt J. (2003). ZNRF proteins constitute a family of presynaptic E3 ubiquitin ligases. *J. Neurosci.* 23 9385–9394. 10.1523/jneurosci.23-28-09385.2003 14561866 PMC6740566

[B4] Araki T. Nagarajan R. Milbrandt J. (2001). Identification of genes induced in peripheral nerve after injury. Expression profiling and novel gene discovery. *J. Biol. Chem.* 276 34131–34141. 10.1074/jbc.m104271200 11427537

[B5] Beerli C. Yakimovich A. Kilcher S. Reynoso G. V. Flaschner G. Muller D. J. (2019). Vaccinia virus hijacks EGFR signalling to enhance virus spread through rapid and directed infected cell motility. *Nat. Microbiol.* 4 216–225. 10.1038/s41564-018-0288-2 30420785 PMC6354922

[B6] Brankatschk B. Wichert S. P. Johnson S. D. Schaad O. Rossner M. J. Gruenberg J. (2012). Regulation of the EGF transcriptional response by endocytic sorting. *Sci. Signal* 5:ra21. 10.1126/scisignal.2002351 22416276

[B7] Chambers M. C. Maclean B. Burke R. Amodei D. Ruderman D. L. Neumann S. (2012). A cross-platform toolkit for mass spectrometry and proteomics. *Nat. Biotechnol.* 30 918–920. 23051804 10.1038/nbt.2377 PMC3471674

[B8] Chang S.-C. Lin W.-L. Chang Y. F. Lee C.-T. Wu J.-S. Hsu P.-H. (2019). Glycoproteomic identification of novel plasma biomarkers for oral cancer. *J. Food Drug Analy.* 27 483–493. 10.1016/j.jfda.2018.12.008 30987719 PMC9296197

[B9] Chattopadhyay S. Veleeparambil M. Poddar D. Abdulkhalek S. Bandyopadhyay S. K. Fensterl V. (2015). EGFR kinase activity is required for TLR4 signaling and the septic shock response. *EMBO Rep.* 16 1535–1547. 10.15252/embr.201540337 26341626 PMC4641505

[B10] Clague M. J. Liu H. Urbe S. (2012). Governance of endocytic trafficking and signaling by reversible ubiquitylation. *Dev. Cell* 23 457–467. 10.1016/j.devcel.2012.08.011 22975321

[B11] de Melker A. A. van der Horst G. Calafat J. Jansen H. Borst J. (2001). c-Cbl ubiquitinates the EGF receptor at the plasma membrane and remains receptor associated throughout the endocytic route. *J. Cell. Sci.* 114 2167–2178. 11493652 10.1242/jcs.114.11.2167

[B12] Demory M. L. Boerner J. L. Davidson R. Faust W. Miyake T. Lee I. (2009). Epidermal growth factor receptor translocation to the mitochondria: regulation and effect. *J. Biol. Chem.* 284 36592–36604. 19840943 10.1074/jbc.M109.000760 PMC2794774

[B13] Du Z. Lovly C. M. (2018). Mechanisms of receptor tyrosine kinase activation in cancer. *Mol. Cancer* 17:58. 10.1186/s12943-018-0782-4 PMC5817791 29455648

[B14] Duan L. Miura Y. Dimri M. Majumder B. Dodge I. L. Reddi A. L. (2003). Cbl-mediated ubiquitinylation is required for lysosomal sorting of epidermal growth factor receptor but is dispensable for endocytosis. *J. Biol. Chem.* 278 28950–28960. 10.1074/jbc.m304474200 12754251

[B15] Eden E. R. Huang F. Sorkin A. Futter C. E. (2012). The role of EGF receptor ubiquitination in regulating its intracellular traffic. *Traffic* 13 329–337. 10.1111/j.1600-0854.2011.01305.x 22017370 PMC3261333

[B16] Elliott G. O’Hare P. (1999). Live-cell analysis of a green fluorescent protein-tagged herpes simplex virus infection. *J. Virol.* 73 4110–4119. 10.1128/jvi.73.5.4110-4119.1999 10196307 PMC104190

[B17] Fan Q. W. Cheng C. Knight Z. A. Haas-Kogan D. Stokoe D. James C. D. (2009). EGFR signals to mTOR through PKC and independently of Akt in glioma. *Sci. Signal* 2:ra4. 10.1126/scisignal.2000014 19176518 PMC2793677

[B18] Goh L. K. Huang F. Kim W. Gygi S. Sorkin A. (2010). Multiple mechanisms collectively regulate clathrin-mediated endocytosis of the epidermal growth factor receptor. *J. Cell. Biol.* 189 871–883. 10.1083/jcb.201001008 20513767 PMC2878939

[B19] Grovdal L. M. Stang E. Sorkin A. Madshus I. H. (2004). Direct interaction of Cbl with pTyr 1045 of the EGF receptor (EGFR) is required to sort the EGFR to lysosomes for degradation. *Exp. Cell. Res.* 300 388–395. 10.1016/j.yexcr.2004.07.003 15475003

[B20] Hallberg B. Rayter S. I. Downward J. (1994). Interaction of Ras and Raf in intact mammalian cells upon extracellular stimulation. *J. Biol. Chem.* 269 3913–3916. 10.1016/s0021-9258(17)41718-2 8307946

[B21] Henne W. M. Buchkovich N. J. Emr S. D. (2011). The ESCRT pathway. *Dev. Cell* 21 77–91. 10.1016/j.devcel.2011.05.015 21763610

[B22] Hirano S. Kawasaki M. Ura H. Kato R. Raiborg C. Stenmark H. (2006). Double-sided ubiquitin binding of Hrs-UIM in endosomal protein sorting. *Nat. Struct. Mol. Biol.* 13 272–277. 10.1038/nsmb1051 16462748

[B23] Huang F. Goh L. K. Sorkin A. (2007). EGF receptor ubiquitination is not necessary for its internalization. *Proc. Natl. Acad. Sci. U. S. A.* 104 16904–16909. 10.1073/pnas.0707416104 17940017 PMC2040475

[B24] Huang F. Kirkpatrick D. Jiang X. Gygi S. Sorkin A. (2006). Differential regulation of EGF receptor internalization and degradation by multiubiquitination within the kinase domain. *Mol. Cell* 21 737–748. 10.1016/j.molcel.2006.02.018 16543144

[B25] Huang F. Zeng X. Kim W. Balasubramani M. Fortian A. Gygi S. P. (2013). Lysine 63-linked polyubiquitination is required for EGF receptor degradation. *Proc. Natl. Acad. Sci. U. S. A.* 110 15722–15727. 10.1073/pnas.1308014110 24019463 PMC3785728

[B26] Janne P. A. Engelman J. A. Johnson B. E. (2005). Epidermal growth factor receptor mutations in non-small-cell lung cancer: implications for treatment and tumor biology. *J. Clin. Oncol.* 23 3227–3234. 15886310 10.1200/JCO.2005.09.985

[B27] Karasneh G. A. Shukla D. (2011). Herpes simplex virus infects most cell types in vitro: clues to its success. *Virol. J.* 8:481. 10.1186/1743-422X-8-481 PMC3223518 22029482

[B28] Kirisits A. Pils D. Krainer M. (2007). Epidermal growth factor receptor degradation: an alternative view of oncogenic pathways. *Int. J. Biochem. Cell. Biol.* 39 2173–2182. 10.1016/j.biocel.2007.07.012 17855153

[B29] Lee C. Y. Lai T. Y. Tsai M. K. Chang Y. C. Ho Y. H. Yu I. S. (2017). The ubiquitin ligase ZNRF1 promotes caveolin-1 ubiquitination and degradation to modulate inflammation. *Nat. Commun.* 8:15502. 10.1038/ncomms15502 PMC5472178 28593998

[B30] Lemmon M. A. Schlessinger J. Ferguson K. M. (2014). The EGFR family: not so prototypical receptor tyrosine kinases. *Cold Spring Harb. Perspect. Biol.* 6:a020768. 10.1101/cshperspect.a020768 24691965 PMC3970421

[B31] Lester J. T. DeLuca N. A. (2011). Herpes simplex virus 1 ICP4 forms complexes with TFIID and mediator in virus-infected cells. *J. Virol.* 85 5733–5744. 10.1128/jvi.00385-11 21450820 PMC3126299

[B32] Levkowitz G. Waterman H. Ettenberg S. A. Katz M. Tsygankov A. Y. Alroy I. (1999). Ubiquitin ligase activity and tyrosine phosphorylation underlie suppression of growth factor signaling by c-Cbl/Sli-1. *Mol. Cell.* 4 1029–1040. 10.1016/s1097-2765(00)80231-2 10635327

[B33] Levkowitz G. Waterman H. Zamir E. Kam Z. Oved S. Langdon W. Y. (1998). c-Cbl/Sli-1 regulates endocytic sorting and ubiquitination of the epidermal growth factor receptor. *Genes Dev.* 12 3663–3674. 10.1101/gad.12.23.3663 9851973 PMC317257

[B34] Lu Q. Hope L. W. Brasch M. Reinhard C. Cohen S. N. (2003). TSG101 interaction with HRS mediates endosomal trafficking and receptor down-regulation. *Proc. Natl. Acad. Sci. U. S. A.* 100 7626–7631. 10.1073/pnas.0932599100 12802020 PMC164637

[B35] Madshus I. H. Stang E. (2009). Internalization and intracellular sorting of the EGF receptor: a model for understanding the mechanisms of receptor trafficking. *J. Cell. Sci.* 122 3433–3439. 10.1242/jcs.050260 19759283

[B36] Raiborg C. Bache K. G. Gillooly D. J. I Madshus H. Stang E. Stenmark H. (2002). Hrs sorts ubiquitinated proteins into clathrin-coated microdomains of early endosomes. *Nat. Cell. Biol.* 4 394–398. 10.1038/ncb791 11988743

[B37] Raiborg C. Stenmark H. (2009). The ESCRT machinery in endosomal sorting of ubiquitylated membrane proteins. *Nature* 458 445–452. 10.1038/nature07961 19325624

[B38] Ravid T. Heidinger J. M. Gee P. Khan E. M. Goldkorn T. (2004). c-Cbl-mediated ubiquitinylation is required for epidermal growth factor receptor exit from the early endosomes. *J. Biol. Chem.* 279 37153–37162. 10.1074/jbc.m403210200 15210722

[B39] Roepstorff K. Grovdal L. Grandal M. Lerdrup M. van Deurs B. (2008). Endocytic downregulation of ErbB receptors: mechanisms and relevance in cancer. *Histochem. Cell. Biol.* 129 563–578. 10.1007/s00418-008-0401-3 18288481 PMC2323030

[B40] Saitoh F. Araki T. (2010). Proteasomal degradation of glutamine synthetase regulates schwann cell differentiation. *J. Neurosci.* 30 1204–1212. 10.1523/jneurosci.3591-09.2010 20107048 PMC6633780

[B41] Sanchala D. S. Bhatt L. K. Prabhavalkar K. S. (2017). Oncolytic Herpes Simplex Viral Therapy: A Stride toward Selective Targeting of Cancer Cells. *Front. Pharmacol.* 8:270. 10.3389/fphar.2017.00270 28559846 PMC5432606

[B42] Schlessinger J. (2002). Ligand-induced, receptor-mediated dimerization and activation of EGF receptor. *Cell* 110 669–672. 10.1016/s0092-8674(02)00966-2 12297041

[B43] Schmidt-Glenewinkel H. Reinz E. Bulashevska S. Beaudouin J. Legewie S. Alonso A. (2012). Multiparametric image analysis reveals role of Caveolin1 in endosomal progression rather than internalization of EGFR. *FEBS Lett.* 586 1179–1189. 10.1016/j.febslet.2012.02.041 22575653

[B44] Smith C. A. Bates P. Rivera-Gonzalez R. Gu B. DeLuca N. A. (1993). ICP4, the major transcriptional regulatory protein of herpes simplex virus type 1, forms a tripartite complex with TATA-binding protein and TFIIB. *J. Virol.* 67 4676–4687. 10.1128/jvi.67.8.4676-4687.1993 8392607 PMC237853

[B45] Smith C. J. Berry D. M. McGlade C. J. (2013). The E3 ubiquitin ligases RNF126 and Rabring7 regulate endosomal sorting of the epidermal growth factor receptor. *J. Cell. Sci.* 126 1366–1380. 10.1242/jcs.116129 23418353

[B46] Sokolowski N. A. Rizos H. Diefenbach R. J. (2015). Oncolytic virotherapy using herpes simplex virus: how far have we come?. *Oncol. Vir.* 4 207–219. 10.2147/ov.s66086 27512683 PMC4918397

[B47] Soltoff S. P. Cantley L. C. (1996). p120cbl is a cytosolic adapter protein that associates with phosphoinositide 3-kinase in response to epidermal growth factor in PC12 and other cells. *J. Biol. Chem.* 271 563–567. 10.1074/jbc.271.1.563 8550620

[B48] Sorkin A. Krolenko S. Kudrjavtceva N. Lazebnik J. Teslenko L. Soderquist A. M. (1991). Recycling of epidermal growth factor-receptor complexes in A431 cells: identification of dual pathways. *J. Cell Biol.* 112 55–63. 10.1083/jcb.112.1.55 1986007 PMC2288797

[B49] Sousa L. P. Lax I. Shen H. Ferguson S. M. De Camilli P. Schlessinger J. (2012). Suppression of EGFR endocytosis by dynamin depletion reveals that EGFR signaling occurs primarily at the plasma membrane. *Proc. Natl. Acad. Sci. U. S. A.* 109 4419–4424. 10.1073/pnas.1200164109 22371560 PMC3311323

[B50] Stang E. Blystad F. D. Kazazic M. Bertelsen V. Brodahl T. Raiborg C. (2004). Cbl-dependent ubiquitination is required for progression of EGF receptors into clathrin-coated pits. *Mol. Biol. Cell.* 15 3591–3604. 10.1091/mbc.e04-01-0041 15194809 PMC491821

[B51] Stang E. Johannessen L. E. Knardal S. L. Madshus I. H. (2000). Polyubiquitination of the epidermal growth factor receptor occurs at the plasma membrane upon ligand-induced activation. *J. Biol. Chem.* 275 13940–13947. 10.1074/jbc.275.18.13940 10788520

[B52] Tomas A. Futter C. E. Eden E. R. (2014). EGF receptor trafficking: consequences for signaling and cancer. *Trends Cell. Biol.* 24 26–34. 10.1016/j.tcb.2013.11.002 24295852 PMC3884125

[B53] Tong J. Taylor P. Moran M. F. (2014). Proteomic analysis of the epidermal growth factor receptor (EGFR) interactome and post-translational modifications associated with receptor endocytosis in response to EGF and stress. *Mol. Cell Proteom.* 13 1644–1658. 10.1074/mcp.m114.038596 24797263 PMC4083106

[B54] Umebayashi K. Stenmark H. Yoshimori T. (2008). Ubc4/5 and c-Cbl continue to ubiquitinate EGF receptor after internalization to facilitate polyubiquitination and degradation. *Mol. Biol. Cell.* 19 3454–3462. 10.1091/mbc.e07-10-0988 18508924 PMC2488299

[B55] Vieira A. V. Lamaze C. Schmid S. L. (1996). Control of EGF receptor signaling by clathrin-mediated endocytosis. *Science* 274 2086–2089. 10.1126/science.274.5295.2086 8953040

[B56] Wahl M. I. Nishibe S. Kim J. W. Kim H. Rhee S. G. Carpenter G. (1990). Identification of two epidermal growth factor-sensitive tyrosine phosphorylation sites of phospholipase C-gamma in intact HSC-1 cells. *J. Biol. Chem.* 265 3944–3948. 10.1016/s0021-9258(19)39685-1 1689311

[B57] Wakatsuki S. Furuno A. Ohshima M. Araki T. (2015). Oxidative stress-dependent phosphorylation activates ZNRF1 to induce neuronal/axonal degeneration. *J. Cell. Biol.* 211 881–896. 10.1083/jcb.201506102 26572622 PMC4657170

[B58] Wakatsuki S. Saitoh F. Araki T. (2011). ZNRF1 promotes Wallerian degeneration by degrading AKT to induce GSK3B-dependent CRMP2 phosphorylation. *Nat. Cell. Biol.* 13 1415–1423. 10.1038/ncb2373 22057101

[B59] Wang Y. Pennock S. Chen X. Wang Z. (2002). Endosomal signaling of epidermal growth factor receptor stimulates signal transduction pathways leading to cell survival. *Mol. Cell. Biol.* 22 7279–7290. 10.1128/mcb.22.20.7279-7290.2002 12242303 PMC139821

[B60] Wang Y. N. Yamaguchi H. Hsu J. M. Hung M. C. (2010). Nuclear trafficking of the epidermal growth factor receptor family membrane proteins. *Oncogene* 29 3997–4006. 10.1038/onc.2010.157 20473332 PMC2904849

[B61] Waterman H. Katz M. Rubin C. Shtiegman K. Lavi S. Elson A. (2002). A mutant EGF-receptor defective in ubiquitylation and endocytosis unveils a role for Grb2 in negative signaling. *EMBO J.* 21 303–313. 10.1093/emboj/21.3.303 11823423 PMC125825

[B62] Yamashita M. Chattopadhyay S. Fensterl V. Saikia P. Wetzel J. L. Sen G. C. (2012). Epidermal growth factor receptor is essential for Toll-like receptor 3 signaling. *Sci. Sign.* 5:ra50. 10.1126/scisignal.2002581 22810896 PMC3431157

[B63] Yang F. C. Tan B. C. Chen W. H. Lin Y. H. Huang J. Y. Chang H. Y. (2013). Reversible acetylation regulates salt-inducible kinase (SIK2) and its function in autophagy. *J. Biol. Chem.* 288 6227–6237. 10.1074/jbc.m112.431239 23322770 PMC3585058

[B64] Zheng K. Xiang Y. Wang X. Wang Q. Zhong M. Wang S. (2014). Epidermal growth factor receptor-PI3K signaling controls cofilin activity to facilitate herpes simplex virus 1 entry into neuronal cells. *MBio* 5 :e00958–13. 24425731 10.1128/mBio.00958-13 PMC3903278

