# Supplementary material for: ZNRF1 Mediates Epidermal Growth Factor Receptor Ubiquitination to Control Receptor Lysosomal Trafficking and Degradation
Source: Front Cell Dev Biol. 2021 Apr 29;9:642625. doi: 10.3389/fcell.2021.642625 (PMC8118649; doi:10.3389/fcell.2021.642625)
Supplement: Supplementary file 1 [file Data_Sheet_1.docx]

Supplementary Material

**Supplementary Figures**

**
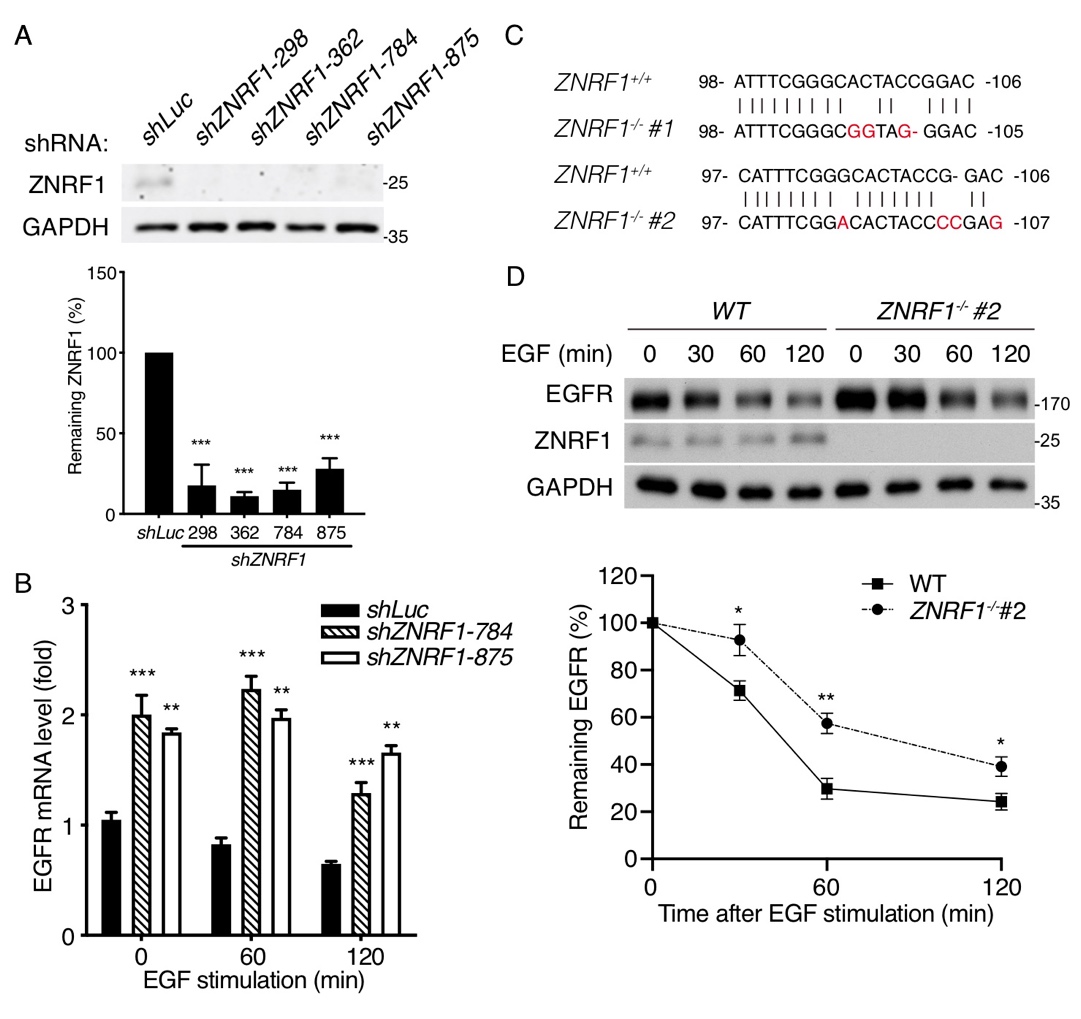

Supplementary Figure 1. Generation of ZNRF1 knockdown and knockout A549 cells.**

(A) A549 cells were infected with lentivirus carrying shRNA against luciferase or four different shRNAs against ZNRF1 for 24 hours, followed by selection with 2 µg/mL puromycin for 72 hours. Cell lysates were collected and ZNRF1 protein levels were examined by immunoblotting. (B) A549 cells expressing *shLuc* and *shZNRF1* were treated with 100 ng/mL EGF for the indicated times. The expression of *EGFR* mRNA was determined by RT-qPCR. (C) Sequence analysis of wild type and two different *ZNRF1*^-/-^ A549 clones generated by the CRISPR/Cas9 system. Genomic DNA from wild type and *ZNRF1*^-/-^ A549 cells was extracted and the region surrounding the targeted site was amplified by PCR for sequencing. Indel mutations are indicated in red. (D) Wild type or *ZNRF1*^-/-^ A549 cells were serum-starved overnight and then stimulated with 100 ng/mL EGF for the indicated times. The levels of EGFR and ZNRF1 proteins were analyzed by immunoblotting. Quantification of immunoblotting analysis data of three independent experiments are shown in the lower panel. Data are presented as mean ± SEM. *P<0.05, **P<0.01, ***P<0.001 (Student’s t-test).

 **Supplementary Figure 2. Overexpression of CAV1 does not affect EGF-induced EGFR degradation.**

(A) Cell lysates from wild type, *ZNRF1*^-/-^, empty vector-, and V5-tagged caveolin-1 (CAV1)-expressing A549 cells were prepared and the level of CAV1 was analyzed by immunoblotting. (B) A549 cells expressing empty vector or V5-tagged caveolin-1 (CAV1) were serum-starved and stimulated with 100 ng/mL EGF for the indicated times. The levels of EGFR and ZNRF1 proteins were analyzed by immunoblotting, and EGFR protein level was quantified as described in Fig. 1. Quantification of immunoblotting analysis data of three independent experiments are shown in the lower panel. Data are presented as mean ± SEM. n.s., no significant; *P<0.05, **P<0.01, ***P<0.001 (Student’s t-test).

**
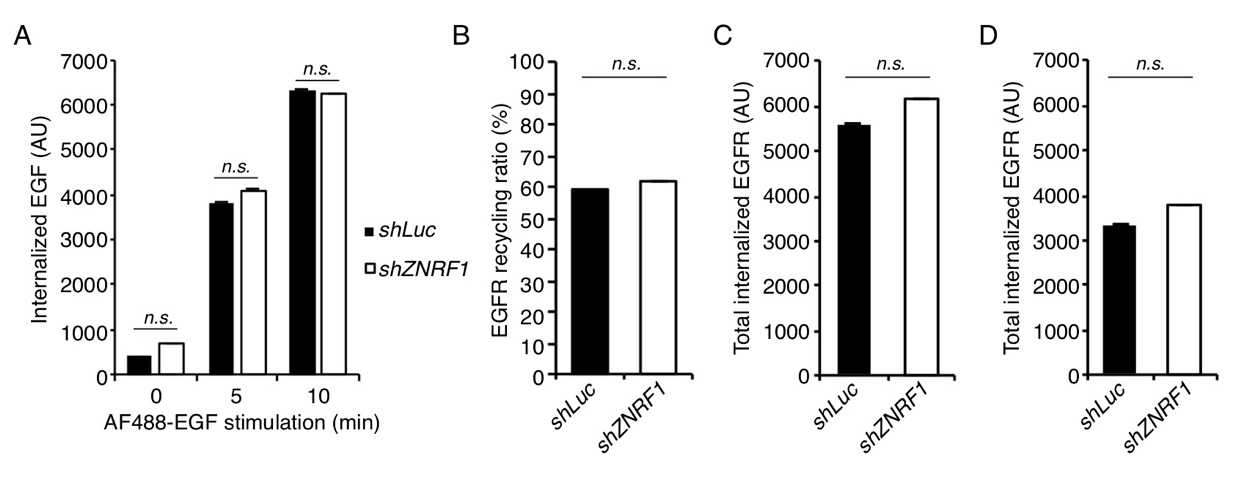

Supplementary Figure 3. Loss of ZNRF1 does not affect EGFR internalization or recycling.**

(A) A549 cells expressing *shLuc* and *shZNRF1* were serum-starved overnight, and then stimulated with 2 µg/mL AF488-conjugated EGF for the indicated times. Cells were harvested, and the amount of internalized EGF-bound EGFR was quantified by flow cytometry. (B-D) The EGFR recycling assay was performed as described in Materials and Methods. (B) The ratio of EGFR recycling (recycled surface EGFR/total EGFR) in *shLuc* and *shZNRF1*-expressing A549 cells. (C) Quantification of total internalized EGFR. (D) Quantification of recycled EGFR 120 minutes after EGF stimulation. Data are presented as mean ± SEM. n.s., no significant; *P<0.05, **P<0.01, ***P<0.001 (Student’s t-test). The data are representative of three independent experiments performed in triplicate.

**Supplementary Figure 4. Loss of ZNRF1 does not alter the interaction of EGFR and CBL.**

HeLa cells expressing *shLuc* or *shZNRF1* shRNA were serum-starved overnight and stimulated with 100 ng/mL EGF for the indicated times. EGFR was immunoprecipitated, and the immunocomplexes as well as total cell lysates (WCL) were subjected to immunoblotting with the indicated antibodies. Quantification of immunoblotting analysis data of three independent experiments are shown in the right panels. Results are presented as averages ± SEM. n.s., no significant; *P<0.05, **P<0.01, ***P<0.001 (Student’s t-test).

**
Supplementary Figure 5. Identification of ZNRF1- and CBL-mediated Ubiquitin-modified lysine residues on EGFR by LC-MS/MS.**

(A and B) HEK293T cells were transfected with Myc-tagged EGFR with empty vector and ZNRF1 (A), or CBL (B) for 48 hours. Cell lysates were prepared and immunoprecipitation with Myc-conjugated agarose, followed by mass spectrometry analysis to identify the ubiquitinated residues on EGFR. Peaks matching expected b and y ions are labeled.
